# Sustainability-oriented recovery of antioxidant and ACE-inhibitory low-molecular-weight peptide fractions from *Thunnus albacares* by-products via enzymatic hydrolysis and membrane ultrafiltration

**DOI:** 10.3389/fnut.2026.1813218

**Published:** 2026-08-21

**Authors:** Mauro Finicelli, Ilenia De Luca, Raffaele Conte, Umberto Galderisi, Mariarosa Anna Beatrice Melone, Gianfranco Peluso, Anna Di Salle

**Affiliations:** 1 Research Institute on Terrestrial Ecosystems (IRET), National Research Council of Italy (CNR), Naples, Italy; 2 IRCCS SYNLAB SDN, Naples, Italy; 3 National Biodiversity Future Center (NBFC), Palermo, Italy; 4 Department of Experimental Medicine, University of Campania "Luigi Vanvitelli", Naples, Italy; 5 2nd Division of Neurology, Department of Advanced Medical and Surgical Sciences, InterUniversity Center for Research in Neurosciences and Center for Rare Diseases, University of Campania "Luigi Vanvitelli", Naples, Italy; 6 Sbarro Institute for Cancer Research and Molecular Medicine, Center for Biotechnology, Temple University, Philadelphia, PA, United States; 7 Faculty of Medicine and Surgery, Saint Camillus International University of Health Sciences, Rome, Italy

**Keywords:** antihypertensive, antioxidant, bioactive peptides, enzymatic hydrolysis, fish by-products, ultrafiltration

## Abstract

**Introduction:**

Tuna by-products are an important and yet underutilized source of bioactive molecules, pointing out the need for innovative blue- and circular-economy-based approaches for their valorization. Fish protein hydrolysate, obtained through enzymatic hydrolysis, are a valuable source of biologically active peptides. Low-molecular-weight (low-MW) peptides derived from fish-byproducts, in addition to their nutritional value, exhibit remarkable health-promoting effects and have a broad potential for industrial applications, including nutraceutical, pharmaceutical, and cosmeceutical.

**Methods:**

In the present study we optimized a valuable strategy that combines enzymatic hydrolysis of tuna mixed by-products including heads, bones, skin, connective tissues, viscera and dark muscle, using Alcalase and Savinase, with ultrafiltration through membranes with molecular weight cut-offs of 10, 5, and 3 kDa. The antioxidant and antihypertensive activities of the resulting peptide fractions were evaluated. The amino acid composition of the most biologically active fractions was subsequently analyzed, and their cytotoxicity was assessed using three *in vitro* cell lines.

**Results:**

Our data identified the low-MW peptide fractions as those with the most prominent antioxidant capacity (AOC) and angiotensin-converting enzyme inhibitory (ACEi) activity, without any observed cytotoxic effect *in vitro*. Notably, the low-MW peptide fraction obtained from Alcalase digestion exhibited the highest bioactive potential (in terms of AOC and ACEi activity at the lowest tested concentration of 12.5 μg/mL) while Savinase-derived low-MW peptide fraction showed greater nutritional value due to its higher content of EAAs. Noteworthy, to the best of our knowledge, this study is among the few to report AOC activity in Savinase-digested tuna by-products.

**Discussion:**

Our results demonstrate that enzymatic hydrolysis combined with membrane ultrafiltration is an effective and sustainability-oriented strategy for recovering bioactive peptide fractions from tuna by-products.

## 1 Introduction

Seafood is an essential component of many diets worldwide (1). Recently, the European Commission estimated that the global population increase by 2050 will lead to a rise in seafood demand up to 60% (2). This is expected to exacerbate the existing challenges such as overfishing, bycatch, species depletion, aquatic pollution, global warming, biodiversity alterations, and acidification of ocean water (3). In line with the blue economic approach, the European nations have introduced novel landing obligations limiting the open water discards. This opens novel opportunities for reducing food loss and for a better management of by-products, such as the skin, bones, connective tissue and viscera (4).

In this scenario, tuna has garnered increasing attention, as it is the most significant marine variety in terms of value and volume of the supply chain, in addition to its notable nutritional importance (5). Evidence indicates that tuna processing leads to approximatively 36% of by-products, mainly consisting of head 17%, skin 8%, viscera 5%, bones 4%, and fins 2%. Tuna by-products are a substantial source of bioactive molecules including vitamins, minerals, polyunsaturated fatty acids, amino acids and peptides (5–7). Innovative approaches aimed at reducing food loss and valorizing tuna by-products into high-value molecules are currently the gold standard for blue- and circular-economy leading to an efficiency of bioresource utilization and a reduction in environmental pollution.

Fish protein hydrolysate, obtained through endogenous or commercial enzymes, are a valuable source of biologically active peptides. This controlled degradation process yields low-molecular-weight peptides that, beyond their nutritional value, exhibit significant health-promoting properties, including antioxidant, anti-inflammatory, antihypertensive, anticancer, and immunoregulatory functions (8–12). Moreover, free peptides derived from the hydrolysis of tuna by-products have a wide range of potential industrial applications. For instance, in nutraceutical formulations, they could enhance the functional, nutritional, and biological properties of the final products (13). Additionally, their antioxidant potential may contribute to improve the physical and chemical stability of these preparations (7, 14). Although enzymatic proteolysis is a suitable approach for obtaining bioactive peptides as functional food ingredients, due to its milder hydrolysis conditions and the production of peptides with diverse sizes and promising biological activities, the optimization of the hydrolysis conditions and the choice of the enzyme still represent critical factors influencing the final yield and quality of bioactive products. Key parameters, such as enzyme type and concentration, pH, hydrolysis time and temperature, as well as the nature of the protein substrate, can significantly affect the production and characteristics of the resulting peptide fractions, including their antioxidant, anti-inflammatory, and antihypertensive potential (15). The existing body of literature indicates that protease from *Bacillus licheniformis* (Alcalase) or protease from *Bacillus* sp. (Savinase) is commonly employed in the enzymatic hydrolysis of fishery by-products. These proteolytic enzymes exhibit substrate specificity that facilitates the generation of distinct peptide profiles. The resulting peptides have been shown to possess varying degrees of bioactivity, particularly in terms of antioxidant potential (16). Comparative studies evaluating the effectiveness of these enzymes against others -such as Neutrase and Papain—on tuna collagen or dark muscle, have demonstrated superior outcomes in terms of peptide yield and associated biological activities (16–18). Thus, this study provides new insights into the effectiveness of Alcalase and Savinase in hydrolyzing a mixed tuna by-products, which have not been extensively investigated compared to individual fractions such as viscera, collagen or dark muscle.

Another key element also influencing the peptide fraction bioactivity is the size; the smaller is the fraction the highest is the bioactive potentiality. Sierra et al. demonstrated that the small size Alcalase hydrolysate (3–10 kDa) of red tilapia (*Oreochromis* sp.) showed good antioxidant activity, probably due to the abundance in amino acids proline, methionine, lysine, phenylalanine, glutamic acid, and aspartic acid (19, 20). Thus, the enzymatic hydrolysis followed by differential ultrafiltration is an effective approach for obtaining peptide fractions of varying molecular sizes, potentially exhibiting distinct biological activities. This strategy is in line with the principles of green chemistry, as it minimizes environmental impact without compromising the efficiency of the reaction. Moreover, it offers a sustainability-oriented alternative to conventional methods used for the recovery of tuna by-products, which often involve hazardous chemicals, higher costs and significant water consumption during the washing and neutralization stages (5).

In the present study we optimized the hydrolysis at mild temperature of *Thunnus albacares* mixed by-products (e.g., bones, skin, and connective tissues) to obtain tuna protein hydrolysates showing the most appropriate degree of hydrolysis (DH). The hydrolysates generated by Alcalase and Savinase were subsequently ultrafiltrated using membranes with molecular weight cut-offs of 10, 5, and 3 kDa. The combination of milder temperature hydrolysis and ultrafiltration supports the more environmentally friendly approach of our study, as the use of *Thunnus albacares* can strengthen the findings from other species (21, 22). The antioxidant and antihypertensive activities of the resulting peptide fractions were assayed. The amino acid composition of the fractions exhibiting the most prominent biological activity were subsequently analyzed and the cytotoxicity assessed in three *in vitro* cell lines.

As already noted, most previous studies have focused on single, isolated fractions, such as viscera, dark muscle, or collagen, leaving the effect of a heterogeneous, industrially representative by-product mixture largely unexplored. Here, we further substantiate this rationale by applying a mild-temperature hydrolysis to a mixed tuna by-product matrix (heads, bones, skin, connective tissue, viscera, and dark muscle), as typically generated by industrial processing, rather than to a single processed fraction. This approach, combined with a direct comparison between Alcalase and Savinase under the same experimental conditions, allowed us to evaluate whether enzyme-specific hydrolysis, together with this compositionally diverse substrate and ultrafiltration procedures, translate into distinct antioxidant and ACE-inhibitory profiles of the resulting low-molecular-weight peptide fractions. Additionally, Alcalase-derived tuna hydrolysates have been repeatedly investigated for their antioxidant potential, comparatively few studies have addressed the bioactivity of Savinase-digested tuna by-products.

## 2 Materials and methods

### 2.1 Materials

*Thunnus albacares* (yellowfin tuna) by-products mixed by-products including heads, bones, skin, connective tissues, viscera and dark muscle were obtained from a local tuna processing industry. The by-product mixture used for each biological replicate was obtained as received from the industrial processing line, reflecting the natural composition and variability of tuna processing waste, without further separation or standardization of individual tissue components. All solvents, reagents, enzymes and standard materials, when not otherwise specified, were purchased from Merck KGaA (Darmstadt, Germany).

### 2.2 Protein extraction and enzymatic hydrolysis

Fish protein extraction was performed as previously described in (13) with a slight modification. Briefly, before the enzymatic hydrolysis, fishery by-products were pre-treated as followed: first the raw material was finely minced using a mixer (IKA-Werke GmbH & Co. KG, Staufen, Germany), then resuspended in H_2_O (1:5 w/v) and sonicated in a water bath at 40 °C for 30 min. The pH of the resulting mixture was adjusted to 8.0 using 1 N NaOH to obtain the optimal reaction conditions for the protease from *Bacillus licheniformis* (Alcalase, ≥2.4 U/g; liquid, Merck KGaA) or protease from *Bacillus* sp. (Savinase, ≥16 U/g; liquid; Merck KGaA). Enzymatic digestion was carried out at 37 °C and pH 8.0 in water bath as previously described (13). The optimal enzyme-to-substrate (E/S) ratios were determined using various E/S ratio (1, 3, and 5% v/w) at predetermined time intervals (every 30 min for 6 h). The E/S ratios of 1, 3, and 5% (v/w) corresponded to 5.3, 16.0, and 26.7 U/g for Alcalase, and 7.7, 23.0, and 38.5 U/g for Savinase, calculated based on the total weight of the mixed by-products, respectively. The enzymes were inactivated by heat treatment (100 °C for 20 min) and the samples centrifuged at 16,000 *g* for 15 min at room temperature (RT) (Ohaus, Switzerland). All the samples were stored at 4 °C until analysis. Three independent batches of the mixed by-product matrix were obtained from the industrial processing line representing three biological replicates. For each batch, hydrolysis reactions with Alcalase and Savinase were performed in duplicates as technical replicates, a control sample without enzyme addition was also included.

### 2.3 Determination of the degree of hydrolysis (DH)

DH was evaluated spectrophotometrically using the method of Borges et al. (23) with slight modifications. Briefly, 50 μL of hydrolyzed samples, were neutralized with 125 μL of 200 mM sodium phosphate buffer (pH 8.2), treated with 50 μL of 0.025% (v/v) 2,4,6-trinitrobenzenesulfonic acid solution (TNBS), and incubated in the dark at 50 °C for 1 h. The reaction was stopped by adding 0.1 M sodium sulfite, cooled down at room temperature, and filtered. The absorbance was measured at 420 nm and α-amino acid was expressed in terms of L-leucine. The DH was determined using the following equation (24):


DH(%)=[(Lt−L0)/(Lmax−L0)]×100

Where *Lt* was the quantity of α-amino acid released at time *t*, *L*0 was the quantity of α-amino acid in the original sample (blank) and *L*max was the maximum quantity of amino groups in fishery by-products. *L*max was obtained by acid hydrolysis of initial sample with 6 M HCl at 105 °C for 24 h. Then, the mixture was filtered using Whatman paper no. 1 and the supernatant neutralized with 6 M NaOH before the amino acids assessment.

### 2.4 Peptide fractions

**SCHEME 1:**
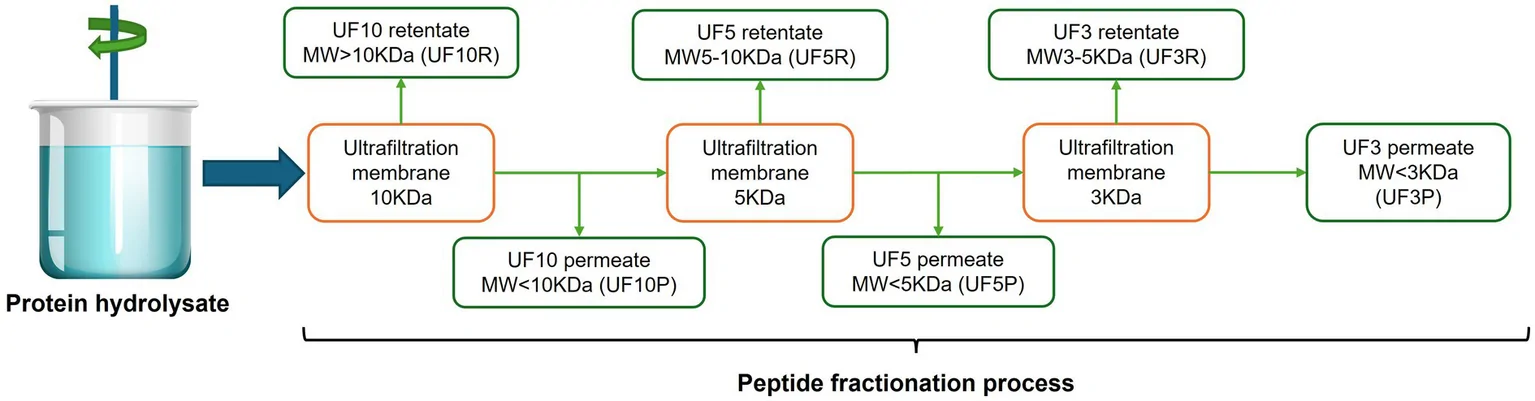
Flow chart of membrane fractionation process.

The peptide fractions were separated using an ultrafiltration technique (Labscale TFF ultrafiltration system, Millipore, Milan, Italy) with sequential 10, 5, and 3 kDa molecular weight cut-off membranes (Millipore, Milan, Italy). Four fractions with different molecular weight were collected for each enzyme: Ultrafiltration retentate with MW > 10 kDa (UF10R), Ultrafiltration retentate with MW 5–10 kDa (UF5R), Ultrafiltration retentate with MW 3–5 kDa (UF3R), Ultrafiltration permeate MW < 3 kDa (UF3P) (Scheme 1). Finally, all fractions were concentrated using SpeedVac™ Vacuum Concentrator (Thermo Fisher Scientific, Italy), freeze-dried and stored at −20 °C until use.

### 2.5 Protein concentration determination of hydrolysates and peptide fractions

Protein concentrations for hydrolysates and peptide fractions were evaluated following the Lowry method (25). The sample absorbances were measured at 500 nm using a microplate reader (Cytation 3, AHSI, Milan, Italy). Bovine serum albumin (BSA) was used as standard.

### 2.6 Antioxidant activity

#### 2.6.1 2,2-diphenyl-1-picrylhydrazyl (DPPH) assay

DPPH radical-scavenging capacity of all peptide fractions was determined as previously reported (26) using Trolox as a standard. Different concentrations of all peptide fractions (ranging from 12.5 to 100 μg/mL) were added to DPPH ethanolic solution (60 μM) and incubated in the dark at 20 °C for 30 min. The absorbance of the samples was determined at 517 nm using a microplate reader (Cytation 3, AHSI, Milan, Italy). The results were expressed as μmol of Trolox equivalent per g of peptide fraction.

#### 2.6.2 2,2-azino-bis-3-ethylbenzothiazoline-6-sulphonic acid (ABTS) radical scavenging activity

The ABTS decolorizations assay was used to determine the free radical scavenging activity of all peptide fractions at different concentrations (ranging from 12.5 to 100 μg/mL) as described by Conte et al. (27) with slight modifications. The ABTS radical cation was generated by reacting ABTS with potassium persulfate. Then, the sample was added to the ABTS solution and incubated in the dark for 20 min. The absorbance was measured at 734 nm using a microplate reader (Cytation 3, AHSI, Milan, Italy). The results were expressed as % of the control (ascorbic acid) at the same concentration.

#### 2.6.3 Ferric reducing antioxidant power (FRAP) assay

FRAP assay, based on the reduction at acidic pH of ferric-tripyridyltriazine (Fe^3+^-TPTZ) was performed according to Henriques et al. (28). 10 μL of peptide fractions at different concentrations (ranging from 12.5 to 100 μg/mL) were placed at 37 °C in a 96-well microplate followed by the addition of 190 μL FRAP reagent and incubated at 37 °C for 5 min. The absorbance was then recorded at 593 nm using a microplate reader (Cytation 3, AHSI, Milan, Italy). A standard curve was obtained with Trolox (0.45–100 μmol/L). The results were expressed as μmol Trolox equivalent (TE) per g of peptide fraction.

### 2.7 Angiotensin-converting-enzyme (ACE) inhibition

The inhibitory effect of peptide fractions on angiotensin-converting enzyme (ACE) was measured using the furanacryloyl tripeptide (FA-Phe-Gly-Gly, FAPGG—Merck) as substrate following the method of Sangsawad et al. (29), with slight modifications. Briefly, 20 μL of different concentrations of peptides (ranging from 12.5 to 100 μg/mL), and 70 μL of 0.5 mM FAPGG in 20 mM phosphate buffer pH 8.3 with 0.1 mM ZnCl_2_, were mixed in a dark 96-well microplate and incubated at 37 °C for 5 min. Then, 10 μL of ACE (peptidyl-dipeptidase A, EC 3.4.15.1—Merck) was added and the absorbance at 340 nm recorded for 60 min using a microplate reader (Cytation 3, AHSI, Milan, Italy). 20 mM phosphate buffer pH 8.3 supplemented with 0.1 mM ZnCl_2_ was used as a positive control. The reaction rate (Δabsorbance/min) was determined for both samples and control and the inhibitory activity was expressed using the following formula:


$$\mathrm{ACE}\;\text{inhibition}\;(\%)=\frac{\text{Slope}\;(\text{positive control})-\text{Slope}\;(\text{sample})}{\text{Slope}\;(\text{positive control})}\times 100$$

### 2.8 Amino acid composition of bioactive peptide fractions

All peptide fractions were hydrolyzed to release their constituent amino acids by adding 6 N hydrochloric acid in a volume ratio of 1:5 and heating the mixture at 110 °C for 20 h (30). Following the reaction, the solution was neutralized to pH 7.0 using a 5% NaOH solution. The resulting sample was diluted to a final volume of 2 mL, and 15 μL of this solution was injected into the ultra-high-performance liquid chromatography (UHPLC) system, coupled with high-resolution mass spectrometry (LCMS8060, Shimadzu Italy, Milan). The instrument parameters were optimized as follows: nebulizing gas flow rate at 2.9 L/min, heating gas flow rate at 10 L/min, interface temperature set to 300 °C, detector temperature at 250 °C, thermal block temperature maintained at 400 °C, and drying gas flow rate at 10 L/min. An internal database created with certified standards (Amino Acid Standard AAS18, Sigma Aldrich) was utilized for the identification of amino acids. The instrument operated with positive electrospray ionization (ESI+) in Multiple Reaction Monitoring (MRM) mode. Compound separation was achieved using a C18 column (3 × 100 mm, 2.6 μm particle size, Phenomenex, Torrance, CA, USA) as the stationary phase, with acetonitrile and water containing 0.01% formic acid as the mobile phase. Instrumental conditions are summarized in Supplementary Table S1.

### 2.9 Peptide fraction cytotoxicity

Human dermal fibroblasts (HDFa; ATCC PCS-201-012), human non-cancer colon fibroblast (CCD-841-CON; ATCC CRL-1790), and human colon epithelial cells, (Caco-2; ATCC HTB-37) were provided from LGC Standards S.r.l. (Sesto San Giovanni, Italy). HDFa and Caco-2 were cultured in Dulbecco’s Modified Eagle Medium (DMEM), while CCD-841-CON in Eagle’s Minimum Essential Medium (EMEM). All cell culture media were supplemented with 1% penicillin, 1% streptomycin and 10% fetal bovine serum (v/v). All cell cultures were maintained at 37 °C in a 5% CO_2_ atmosphere. To evaluate fish waste-derived peptide fraction cytotoxicity, a Cell Counting Kit-8 (CCK-8) assay was carried out. Briefly, all cell lines were seeded in a 96-well plate at a density of 4 × 10^3^ cells/well. After 24 h, the cells were treated with 10 μL of peptide fractions at different concentrations (12.5–100 μg/mL) and incubated at 37 °C for 24, 48 and 72 h. Then, 10 μL of CCK-8 solution were added to each well, and the plate was incubated under cell culture conditions for 1–4 h. The absorbance at 450 nm was measured using a microplate reader (Cytation 3, AHSI, Milan, Italy).

### 2.10 Statistical analysis

All quantitative data are expressed as the mean ± standard deviation (SD) using GraphPad Prism 6 software (GraphPad Software Inc., San Diego, CA, USA) to statistically compared the different experimental groups and controls. One-way ANOVA was used for DH, two-way ANOVA with Tukey’s multiple comparison test for AOC, ACE inhibitory assays, and cytotoxicity test. Unpaired t test with Welch correction and two-stage step-up (31) method for multiple comparison was used for amino acid analysis. EC50 values were calculated for the DPPH and ABTS assays by non-linear regression (four-parameter logistic model; GraphPad Prism 6).

## 3 Results

### 3.1 Hydrolysis and peptide fractionation

**Figure 1:**
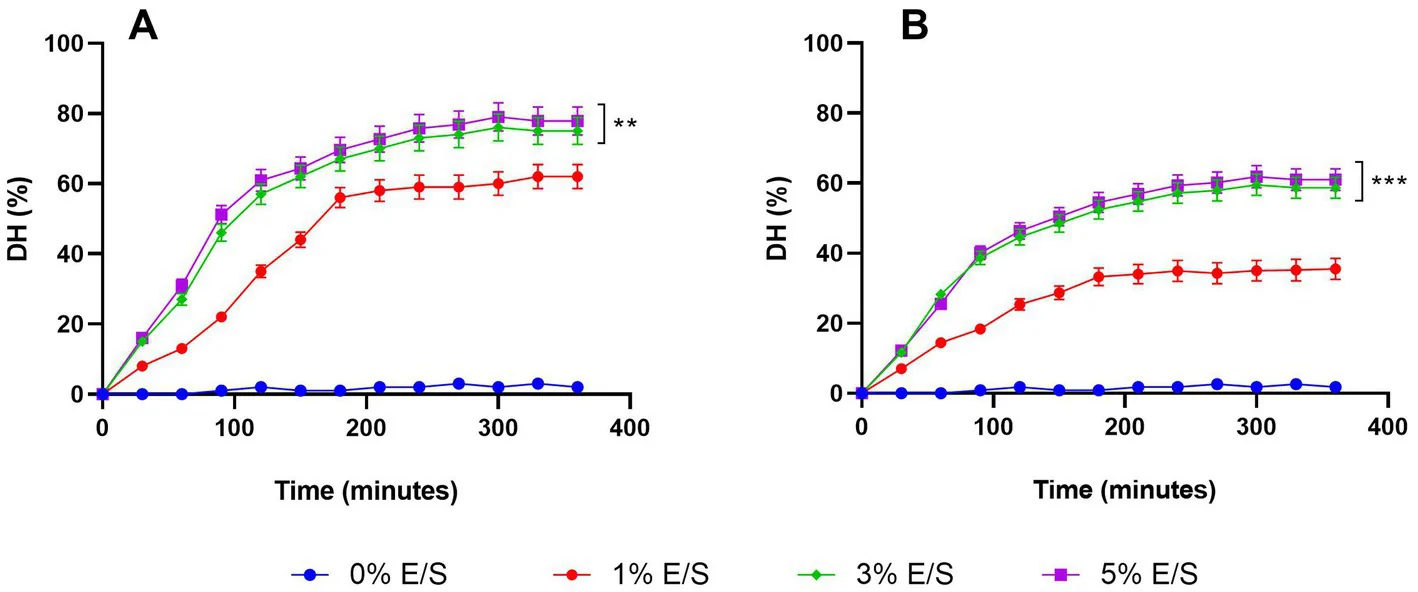
Degree of hydrolysis obtained from fish by-products with **(A)** Alcalase, and **(B)** Savinase. The bars represent means ± SD (*n* = 6). Statistically significant variations: ^**^p < 0.01, and ^***^p < 0.001 versus 1% E/S.

The homogenized tuna by-products were hydrolyzed using two types of proteases, frequently used to achieve enzymatic hydrolysis of fish by-products, namely Alcalase, and Savinase (16, 32–34). The reaction was performed at 37 °C and the efficiency of the process was assessed over 6 h by determining the degree of hydrolysis (DH) using different enzyme/substrate (E/S) ratios. As shown in Figure 1, the hydrolysis process for both enzymes demonstrated a rapid initial phase within almost the first 2 h, followed by a slower progression reaching a plateau. The increase in enzyme concentration from 1% to 3 and 5% resulted in a statistically significant increase in DH for both enzymes (^**^p < 0.01, and ^***^p < 0.001 vs. 1% E/S for Alc and Sav, respectively). No differences were observed between 3 and 5% E/S for both Alcalase or Savinase.

Alcalase exhibits a higher reaction rate reaching after 120 min a DH of 61 and 57% for the E/S ratio of 5 and 3%, respectively, while Savinase proceeds more slowly reaching only after 150 min a DH of 50 and 48% for E/S ratio 5 and 3%, respectively. According to these data, the optimal hydrolysis conditions in terms of minor enzyme concentration and reaction time were: Alcalase 3% E/S, 120 min (Alc-H); Savinase 3% E/S, 150 min (Sav-H).

Total hydrolyzed fractions, Alc-H and Sav-H, were subjected to the ultrafiltration process to achieve peptide separation by using 10, 5 and 3 kDa ultrafiltration membranes. As reported in Scheme 1, 4 peptide fractions for each enzyme, namely Alc-UF10R, Alc-UF5R, Alc-UF3R, Alc-UF3P, and Sav-UF10R, Sav-UF5R, Sav-UF3R, Sav-UF3P, were obtained.

Table 1 summarizes the protein/peptide concentration in all hydrolyzed and peptide fractions.

**Table 1:** Protein concentration (mg/mL) of hydrolysates and peptide fractions.

Alcalase			Savinase		
Sample	Protein concentration (mg/mL)	*p* value	Sample	Protein concentration (mg/mL)	*p* value
Alc-H	1.66 ± 0.08	–	Sav-H	1.29 ± 0.07	–
Alc-UF10R	1.54 ± 0.05	0.0923	Sav-UF10R	1.74 ± 0.06	**0.0011**
Alc-UF5R	1.21 ± 0.06	**0.0015**	Sav-UF5R	1.11 ± 0.04	**0.018**
Alc-UF3R	0.69 ± 0.01	**<0.0001**	Sav-UF3R	0.44 ± 0.01	**<0.0001**
Alc-UF3P	0.45 ± 0.01	**<0.0001**	Sav-UF3P	0.28 ± 0.01	**<0.0001**

Bold values indicate statistically significant differences (*p* < 0.05).

### 3.2 Antioxidant activity

Complementary approaches were employed to evaluate the antioxidant capacity (AOC) of all hydrolysates and peptide fractions, aiming to investigate the different chemical reaction mechanisms involved. All samples were analyzed at various concentrations (12.5, 25, 50, and 100 μg/mL) to assess their free radical scavenging abilities (using DPPH and ABTS assays) and their ferric ion-reducing capacity (using the FRAP assay). DPPH and ABTS assays are widely used to measure the radical scavenging activity of peptides, phenolic compounds, and food matrices (35), whereas the FRAP assay assesses the reduction of ferric ions (Fe^3+^) to ferrous ions (Fe^2+^), thus providing an estimation of the reducing power of the sample.

**Figure 2:**
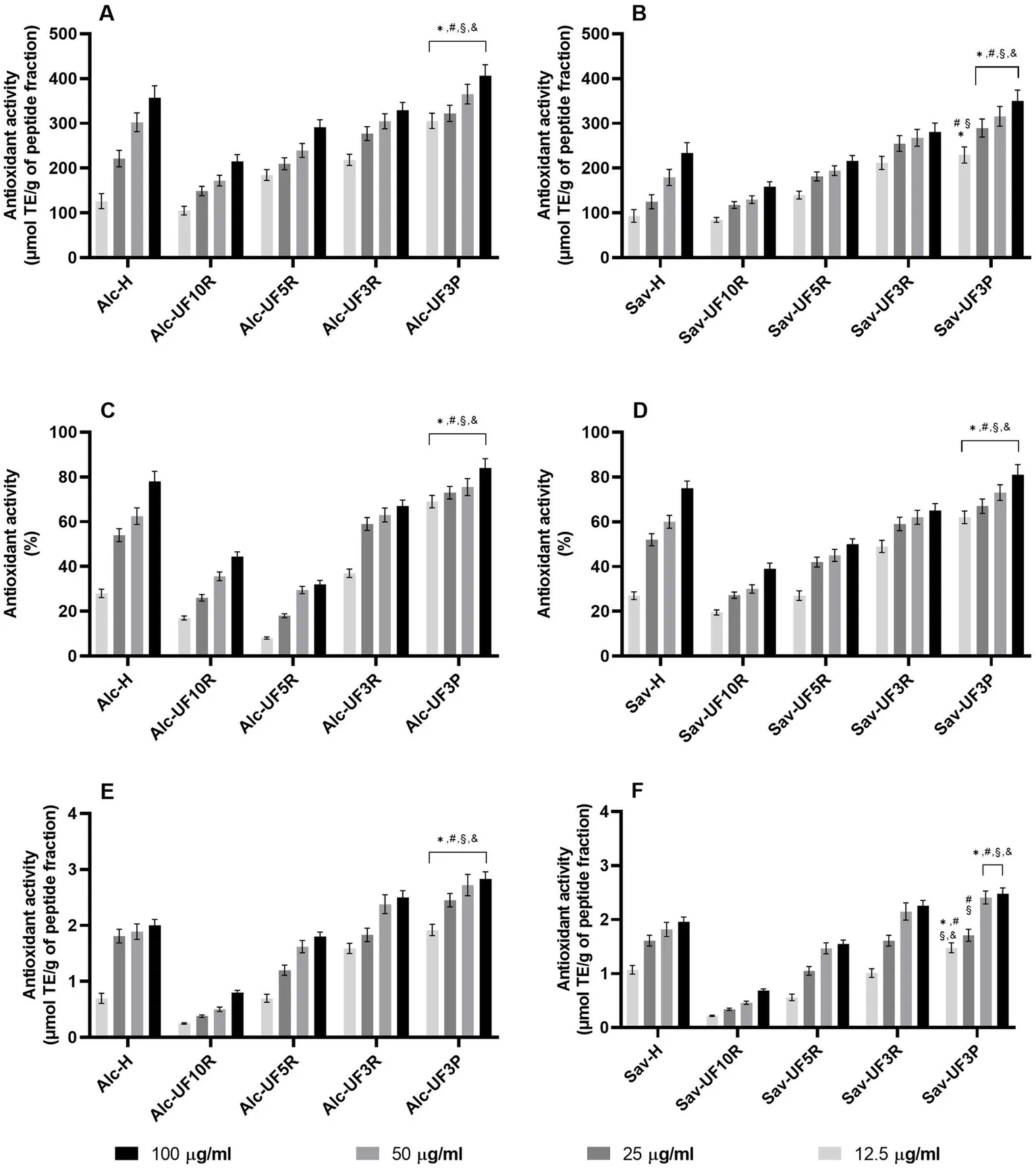
DPPH, ABTS radical scavenging activity and FRAP activity of hydrolyzed and peptide fractions obtained with Alcalase **(A,C,E)** and Savinase **(B,D,F)**. The bars represent means ± SD (*n* = 6). Statistical significance was determined using two-way ANOVA: ^*^UF3P vs. H; ^#^UF3P vs. UF10R; ^§^UF3P vs. UF5R; ^&^UF3P vs. UF3R. The *p*-values are listed in Supplementary Tables S2–S7.

As shown in Figure 2, low-molecular-weight peptide fractions generally exhibited higher AOC across all tested concentrations than both the total hydrolysates and the high-molecular-weight fractions. Because each assay was performed on multiple peptide fractions and concentrations, and the corresponding statistical analyses are extensive, the full statistical outcomes are reported in Supplementary Tables S2–S7. Moreover, a linear increase in both antioxidant capacity and reducing power was observed with increasing peptide concentration for all tested samples.

**Figure 3:**
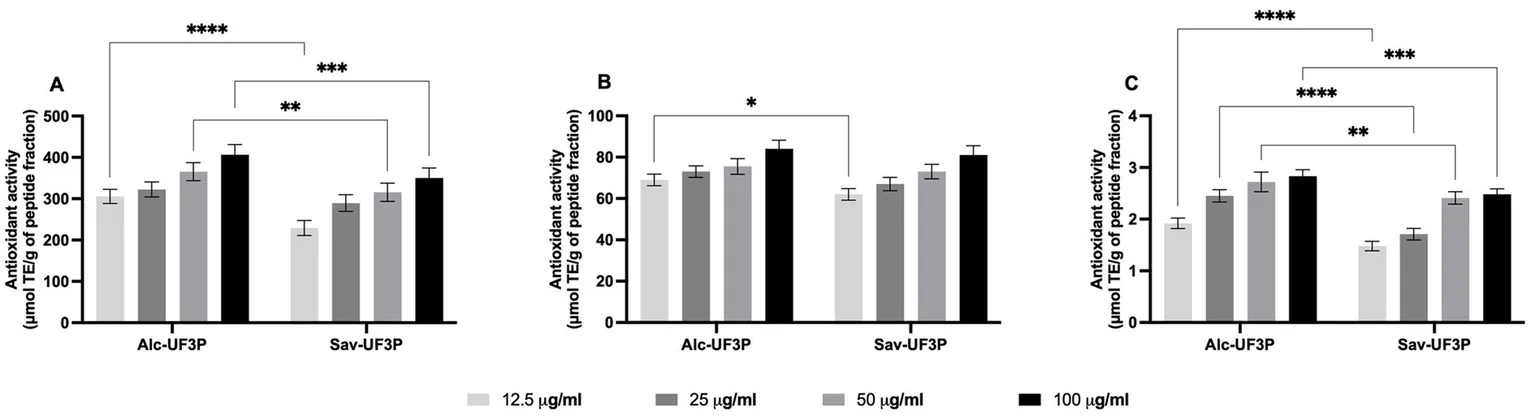
Comparison of the antioxidant capacity of Alc-UF3P and Sav-UF3P fractions **(A)** DPPH, **(B)** ABTS, and **(C)** FRAP. Results are expressed as μmol Trolox equivalents/g of peptide fractions and represent the mean ± standard deviation (*n* = 6). Statistical significance: ^*^p ≤ 0.05, ^**^p ≤ 0.01, ^***^p ≤ 0.001 and ^****^p ≤ 0.0001.

Notably, the Alc-UF3P peptide fraction demonstrated a significantly greater (Figure 3) antioxidant capacity compared to the corresponding Savinase-derived fraction, already at the lowest tested concentration of 12.5 μg/mL (*p* ≤ 0.0001 for DPPH and FRAP assays, and *p* ≤ 0.05 for ABTS assay). At higher concentrations, statistical significance was maintained for the DPPH and FRAP assays, whereas the ABTS assay demonstrated only a qualitative dose–response trend.

To further quantify the antioxidant potency of the peptide fractions, EC50 values were calculated for the DPPH and ABTS assays by non-linear regression and are reported, together with their 95% confidence intervals and goodness of fit (*R*^2^), in Table 2. Overall, EC50 values decreased progressively with decreasing molecular weight for both enzymes, mirroring the increase in antioxidant activity described above. For the most bioactive fractions (Alc-UF3P, and Sav-UF3P), the estimated EC50 fell below the lowest tested concentration (12.5 μg/mL). These values, although associated with relatively narrow confidence intervals, should be regarded as extrapolated estimates, indicating that these fractions retain substantial antioxidant activity even below the tested concentration range. For the Alc-UF5R fraction (DPPH assay), the dose–response curve showed a markedly shallower slope compared to all other fractions, resulting in a poorly constrained EC50 estimate with a correspondingly wide 95% confidence interval (Table 2).

**Table 2:** EC50 values (with 95% CI and goodness of fit) for DPPH and ABTS radical scavenging activity of tuna by-product hydrolysates and peptide fractions.

Sample	EC50 DPPH (μg/mL)	95% CI DPPH	*R*^2^ DPPH	EC50 ABTS (μg/mL)	95% CI ABTS	*R*^2^ ABTS
Alc-H	19.39	17.41–21.84	0.976	18.82	16.94–21.36	0.981
Alc-UF10R	15.62	12.96–22.03	0.973	21.15	19.01–24.13	0.986
Alc-UF5R	13.52^b^	8.36–5998ᵇ	0.976	21.72	20.48–23.06	0.987
Alc-UF3R	7.75^a^	6.10–9.15	0.984	11.41^a^	10.60–12.11	0.989
Alc-UF3P	4.95^a^	3.06–6.78	0.973	2.67^a^	1.21–4.37	0.982
Sav-H	25.06	19.58–42.84	0.953	18.04	16.47–19.97	0.984
Sav-UF10R	11.64^a^	9.89–13.26	0.969	18.04	13.86–39.30	0.975
Sav-UF5R	7.92^a^	6.22–9.38	0.982	11.33^a^	10.21–12.32	0.984
Sav-UF3R	6.02^a^	2.93–8.51	0.973	5.89^a^	3.71–7.74	0.986
Sav-UF3P	7.98^a^	6.10–9.60	0.972	4.46^a^	2.83–6.08	0.981

a Estimated value below the lowest tested concentration (12.5 μg/mL); extrapolated estimate.

b EC50 poorly constrained due to a shallow dose–response curve (Hill slope = 0.55); the extremely wide 95% CI reflects this limited precision and the value should be interpreted qualitatively only.

### 3.3 Angiotensin-converting-enzyme (ACE) inhibition

In addition to antioxidant activity, the ACE inhibitory (ACEi) potential of the hydrolysates and peptide fractions was also evaluated, considering the importance of multifunctional peptides with both antihypertensive and antioxidant properties.

**Figure 4:**
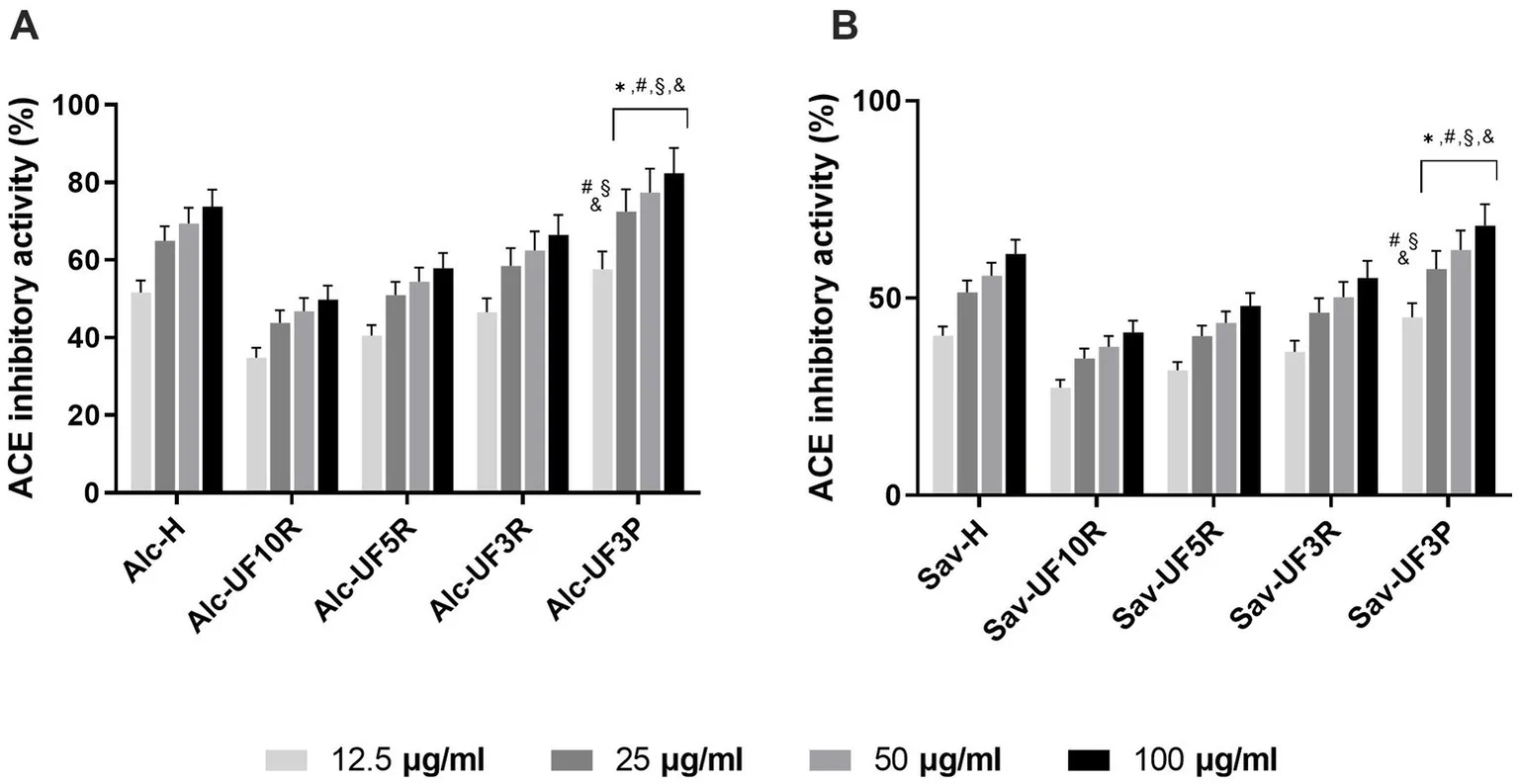
ACEi activity of hydrolyzed and peptide fractions obtained with Alcalase **(A)** and Savinase **(B)**. The inhibitory activity was expressed as % respect to 0.1 mM ZnCl_2_ used as positive control. The bars represent means ± SD (*n* = 6). Statistical significance was determined using two-way ANOVA: ^*^UF3P vs. H; ^#^UF3P vs. UF10R; ^§^UF3P vs. UF5R; ^&^UF3P vs. UF3R. The *p*-values are listed in Supplementary Tables S8–S9.

Each peptide fraction was assessed for its ACEi activity (%) as shown in Figure 4.

**Figure 5:**
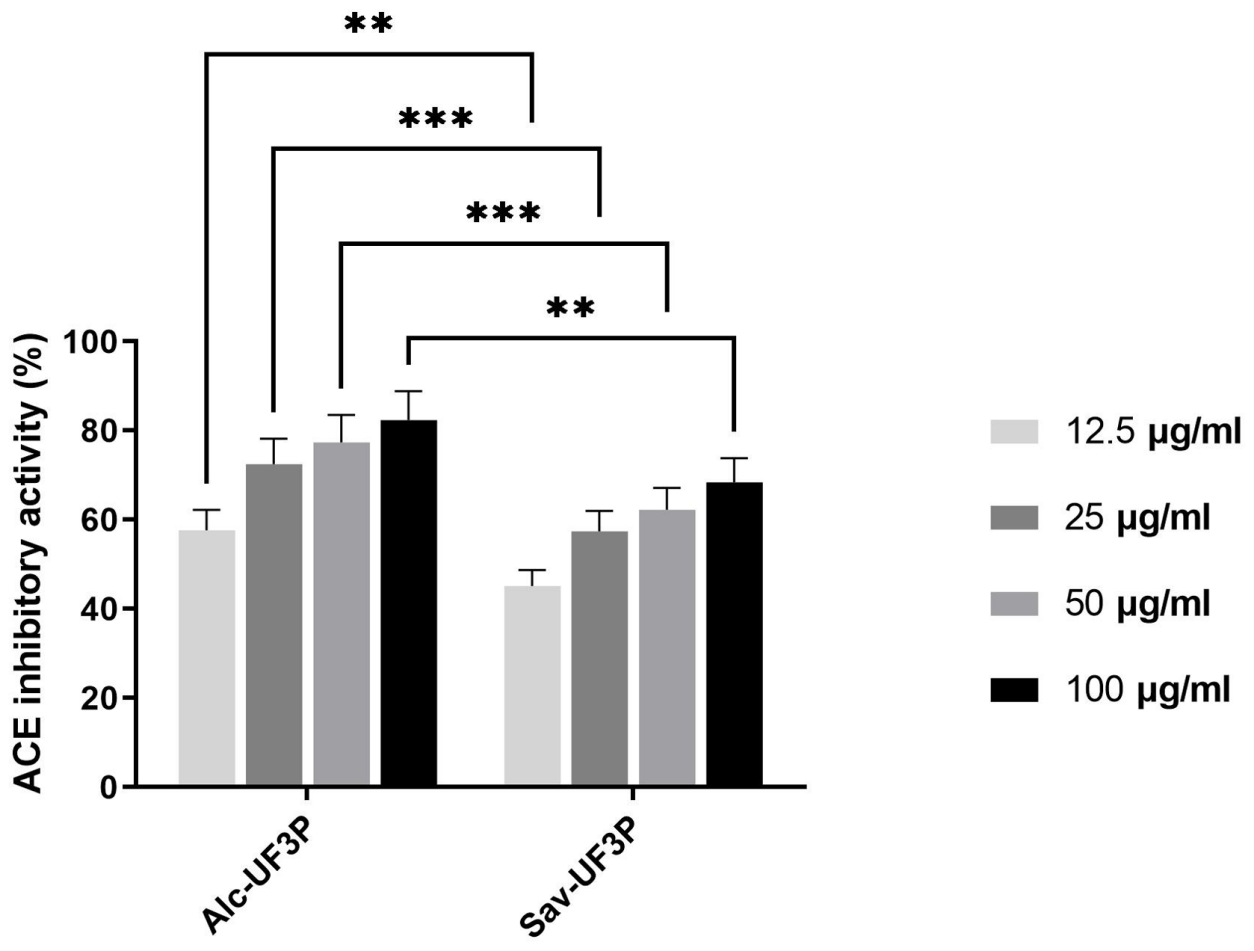
Comparison of the ACEi activity of Alc-UF3P and Sav-UF3P fractions. Results are expressed as % respect to 0.1 mM ZnCl_2_ used as a positive control and represent the mean ± standard deviation (*n* = 6). Statistical significance was determined using two-way ANOVA; ^**^p ≤ 0.01, and ^***^p ≤ 0.001.

Overall, the low molecular weight peptide fraction obtained through Alcalase digestion demonstrated significantly higher inhibitory activity compared to the fraction generated by Savinase, across all tested concentrations (Figure 5).

Furthermore, the results revealed a clear inverse relationship between the molecular weight of the peptide fractions and their ACEi capacity, with the highest inhibition observed for the Alc-UF3P fraction at a concentration of 100 μg/mL (82.34 ± 6.56%).

Interestingly, this same Alcalase-derived fraction already exhibited a substantial ACEi activity at a concentration of 12.5 μg/mL (57.6 ± 4.6%). In contrast, the low-molecular-weight fraction obtained through Savinase digestion (Sav-UF3P) demonstrated an inhibition rate of 68.3 ± 5.4% only at 100 μg/mL, thus indicating a lower efficacy compared to the corresponding Alcalase fraction.

### 3.4 Analysis of amino acids

Liquid chromatography–mass spectrometry was used to determine the amino acid profile of the most bioactive peptide fractions, Alc-UF3P and Sav-UF3P (Table 3). Moreover, the abundance of essential amino acids (EEAs—Histidine, Phenylalanine, Isoleucine, Lysine, Leucine, Methionine, Threonine, Tryptophan, and Valine) in both peptide fractions was also evaluated.

**Table 3:** Amino acid composition (expressed as relative percentage) of two peptide fractions, determined by LC–MS analysis.

Amino acid	Alc-UF3P (%)	Sav-UF3P (%)	Two-tailed *p* value	Statistically significant difference
A (alanine)	9.22 ± 0.53	8.65 ± 0.62	0.29	n.s.
C (cysteine)	6.00 ± 0.34	5.56 ± 0.32	0.18	n.s.
D (aspartic acid)	10.56 ± 0.67	6.21 ± 0.41	0.002	^**^
E (glutamic acid)	10.48 ± 0.67	3.95 ± 0.24	0.001	^***^
F (phenylalanine)	6.45 ± 0.46	7.26 ± 0.45	0.10	n.s.
G (glycine)	9.28 ± 0.55	7.73 ± 0.49	0.02	^*^
H (histidine)	0.86 ± 0.05	1.56 ± 0.11	0.003	^**^
I (isoleucine)	6.17 ± 0.34	7.86 ± 0.39	0.005	^**^
K (lysine)	1.32 ± 0.08	4.86 ± 0.29	0.001	^***^
L (leucine)	10.06 ± 0.79	9.04 ± 0.62	0.16	n.s.
M (methionine)	0.92 ± 0.08	0.82 ± 0.05	0.14	n.s.
N (asparagine)	3.05 ± 0.19	3.13 ± 0.17	0.61	n.s.
P (proline)	8.41 ± 0.56	6.34 ± 0.29	0.01	^**^
Q (glutamine)	1.84 ± 0.10	3.26 ± 0.22	0.002	^**^
R (arginine)	1.46 ± 0.09	4.49 ± 0.25	<0.001	^***^
S (serine)	1.98 ± 0.14	2.26 ± 0.12	0.06	n.s.
T (threonine)	3.83 ± 0.20	5.08 ± 0.33	0.008	^**^
V (valine)	6.21 ± 0.39	8.79 ± 0.47	0.002	^**^
W (tryptophane)	0.80 ± 0.04	1.56 ± 0.11	0.003	^**^
Y (tyrosine)	1.10 ± 0.09	1.59 ± 0.11	0.004	^**^
EAAs	36.62 ± 0.27	46.83 ± 0.31	<0.001	^***^

EAAs, essential amino acids. Data are presented as mean ± standard deviation (*n* = 6). Statistical differences between the two fractions were assessed using unpaired *t* test with Welch correction and two-stage step-up (Benjamini, Krieger, and Yekutieli) method for multiple comparison: ^*^p ≤ 0.05, ^**^p ≤ 0.01, ^***^p ≤ 0.001.

The amino acid profiles of the Alc-UF3P and Sav-UF3P peptide fractions exhibited notable qualitative similarities reflecting the correspondences highlighted in their bioactivity, although quantitative differences in specific residues were present (Table 3).

Both fractions were rich in aliphatic and acidic amino acids; however, the Alc-UF3P showed a significantly higher abundance of aspartic acid (10.56% vs. 6.21%, *p* ≤ 0.001), glutamic acid (10.48% vs. 3.95%, *p* ≤ 0.0001), and glycine (9.28% vs. 7.73%, *p* ≤ 0.01) compared to the Sav-UF3P. Aromatic residues such as phenylalanine and tryptophan were more prominent in Sav-UF3P (7.26 and 1.56%, respectively) than in Alc-UF3P (6.45 and 0.80%), indicating a higher hydrophobic character in the Savinase-derived fraction. The basic amino acids lysine and arginine were significantly elevated in Sav-UF3P (4.86 and 4.49%, respectively) relative to Alc-UF3P (1.32 and 1.46%) (*p* ≤ 0.001), potentially contributing to differences in net charge and bioactivity. Hydrophobic branched-chain amino acids (Ile, Leu, Val) were present at comparable or higher levels in Sav-UF3P, particularly isoleucine (7.86% in Sav-UF3P vs. 6.17% in Alc-UF3P, *p* ≤ 0.01) and valine (8.79% in Sav-UF3P vs. 6.21% in Alc-UF3P, *p* ≤ 0.01). Conversely, proline was significantly more abundant in Alc-UF3P (8.41%) than in Sav-UF3P (6.34%) (*p* ≤ 0.01).

Regarding EEAs, the results showed that Savinase digestion led to a statistically significant increase (*p* ≤ 0.001) in EEAs content in the Sav-UF3P fraction compared to the Alc-UF3P fraction.

### 3.5 Peptide fractions cytotoxicity

**Figure 6:**
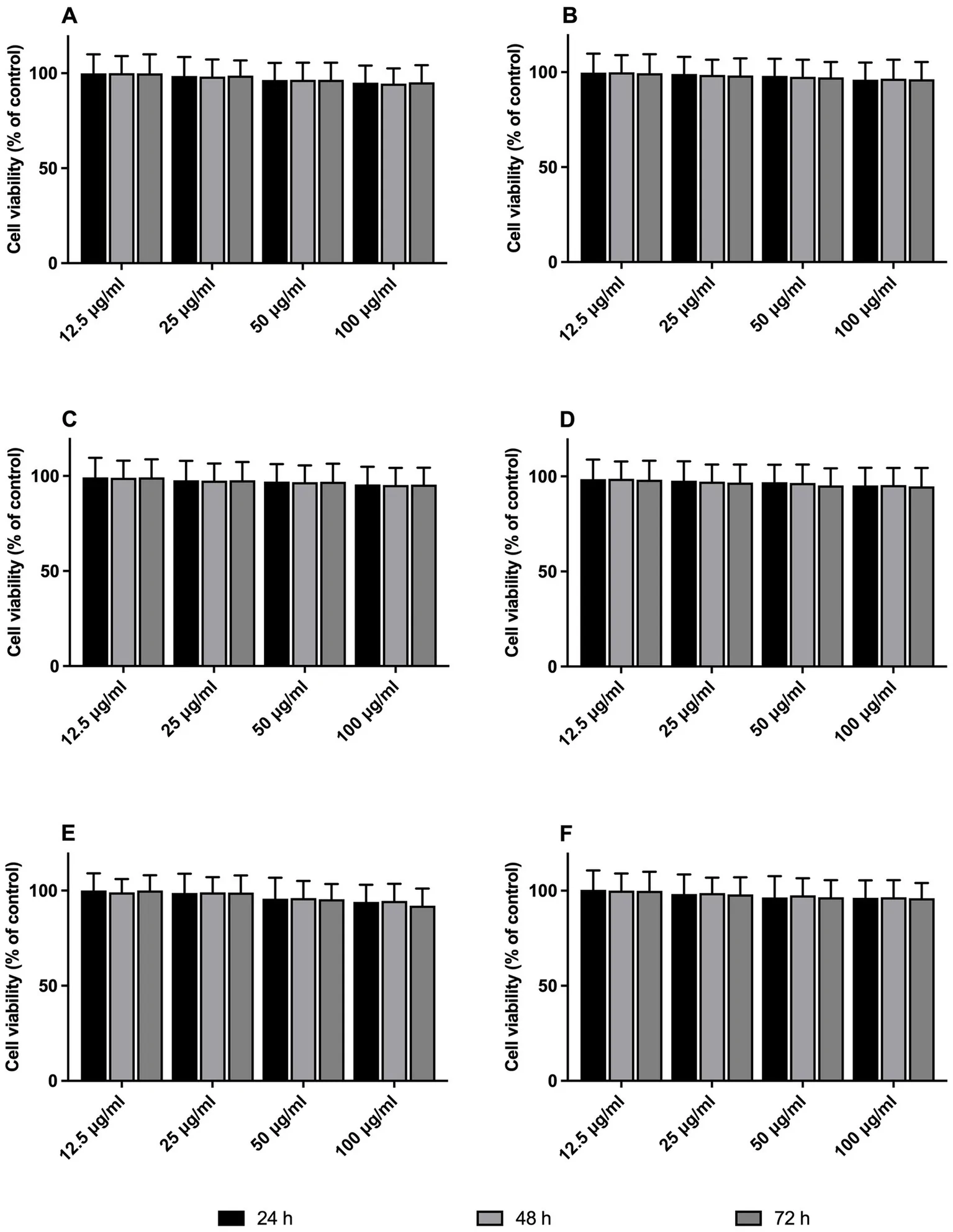
*In vitro* biocompatibility of Alc-UF3P and Sav-UF3P. Cytotoxicity was evaluated in HDFs **(A,B)** for Alc-UF3P and Sav-UF3P, CCD-841-CoN cells **(C,D)**, and Caco-2 cells **(E,F)** following 24, 48, and 72 h of incubation with peptide fractions at concentrations of 12.5, 25, 50, and 100 μg/mL.

Cytotoxicity of the bioactive peptide fractions was evaluated using the CCK-8 assay at various concentrations in three different cell lines: human dermal fibroblasts (HDFa), human non-cancerous colon fibroblasts (CCD-841-CoN), and human colon epithelial cells (Caco-2). Cells were incubated with peptide fractions for 24, 48, and 72 h. As shown in Figure 6, neither Alc-UF3P nor Sav-UF3P exhibited any cytotoxic effects on the tested cell lines, even after 72 h of incubation, at concentrations of 12.5, 25, 50, and 100 μg/mL.

## 4 Discussion

In the present study, we propose a strategy that combines enzymatic hydrolysis of tuna by-products with membrane ultrafiltration to obtain peptide fractions with potential bioactive properties, addressing the ongoing need for sustainability-oriented valorization strategies within the seafood processing chain (36). Among the different proteolytic enzymes commonly used to hydrolyze fish proteins, in the present study we used Alcalase and Savinase, two serine endoproteinases working at alkaline conditions. Devita and colleagues demonstrated the effectiveness of these enzymes in producing hydrolysates from tuna collagen showing bioactive properties (16). We adopted a milder hydrolysis temperature compared to conditions commonly reported in recent literature, to preserve the stability of both the enzymes and the resulting peptides during the incubation times. These experimental conditions were reported and validated in our previous study (13). This approach was also intended to minimize the occurrence of secondary reactions that could negatively affect the integrity and bioactivity of peptides within the hydrolysate. Finally, this choice supports the more environmentally friendly approach of our study, as the use of milder temperature reduces energy consumption for potential industrial scale-up. Our data showed that both the enzymes were able to hydrolyze proteins from tuna by-products. Moreover, an increase in enzyme concentration from 1 to 5% of E/S leads to a proportional higher DH. This finding is consistent with the body of literature reporting the efficacy of these enzymes (16, 23) and also demonstrates that milder temperature did not affect the DH of either enzyme. Notably, both Alcalase and Savinase showed a high rate of hydrolysis within the first 100 min followed by a slower progression reaching a plateau. This behavior is consistent with the kinetics of the classical hydrolysis reaction, characterized by a rapid initial phase, during which the enzymes cleave the peptide bonds, along with a stationary state due to the lower availability of the remaining bonds (37). Overall, these data indicate that we setup enzymatic digestions of tuna by-products with a high and efficient DH. Alcalase 3% E/S, 120 min (Alc-H) and Savinase 3% E/S, 150 min (Sav-H) resulted the optimal hydrolysis conditions, balancing minimal enzyme input with effective reaction time. The high DH values obtained (61% for Alcalase and 50% for Savinase in the optimal reaction conditions) can be the result of three main factors: (i) the broad substrate specificity of the selected serine-endopeptidases, (ii) the heterogeneity of the starting material (mixed by-products including heads, bones, skin, connective tissues, viscera and dark muscle), and (iii) the E/S ratios employed. Alcalase and Savinase show distinct specificity: Alcalase preferentially cleaves at P1 positions occupied by Glu, Asp., Lys, Leu, and Tyr, while Savinase prefers hydrophobic residues (Phe, Ile, Val), and basic residues (38, 39). These specificities are directly reflected in our amino acid profiles: Alc-UF3P was enriched in Glu (10.48%) and Asp (10.56%), while Sav-UF3P showed higher abundance of Phe (7.26%), Lys (4.86%), Ile (7.86%), and Val (8.79%). We can hypothesize that our heterogeneous substrate provided a higher density of diverse cleavage sites compared to more restricted substrate, such as isolated viscera (23, 40), dark muscle alone (18), tuna head fractions (41), or collagen-enriched fractions (16, 42, 43), commonly reported in studies with lower DH values. Taken together, the combination of these factors with the high E/S ratio used accounts for the elevated DH observed in both hydrolysis reactions, even when operating at the mild temperature of 37 °C.

Although the optimization of hydrolysis condition is a crucial point to producing desirable bioactive protein hydrolysate, some concerns still remain about the effectiveness of the crude hydrolysate. Indeed, the crude hydrolysate solution is a mixture of different sized peptides characterized by bioactive and non-bioactive properties. Membrane ultrafiltration allows isolation of bioactive mixtures by molecular weight, offering more environmentally friendly alternative to the use of toxic and expensive solvents required by other separation approaches, e.g., chromatography (44–46). Moreover, several studies have used ultrafiltration to obtain peptide fractions showing antioxidants and hypertensive activities (46, 47).

Looking at the protein concentrations of the peptide fractions obtained from the ultrafiltration process, we observed that the differences between the Alcalase and Savinase fractions were more pronounced at lower molecular weights (Alc-UF3R and Alc-UF3P vs. Sav-UF3R and Sav-UF3P, see Table 1). This observation, together with the higher DH achieved by Alcalase, suggests that Alcalase hydrolysis may be more effective in yielding low-molecular-weight peptides. Consistent with this, Alc-UF3P and Sav-UF3P showed the highest antioxidant activity across all assays and concentrations tested, given that peptide molecular weight strongly influences bioactivity (46, 48–53). To verify this hypothesis, we investigated the AOC potentials of the crude hydrolysate and the ultrafiltered fractions at different concentrations. We used three different assays to demonstrate the AOC of our samples. DPPH and ABTS assays tested the free radical scavenging abilities while and the FRAP assay established the ferric ion-reducing capacity. Although the crude hydrolysate for Alcalase and Savinase showed a marked AOC for all the assays, the differences in peptide MW distributions seemed to be effective in explaining the changes in antioxidant properties of the ultrafiltered fractions. Indeed, Alc-UF3P and Sav-UF3P showed the higher AOC for all the concentration tested in all the assays tested.

Yellowfin tuna peptides showed a marked increase in antioxidant and anti-microbial activities when the lowest molecular weight fraction was assayed (46). Thus, our data are in line with the finding that the MW of the peptides influences the antioxidant activity (50). Our data are consistent with those obtained from fish by-product hydrolysate, showing that ultrafiltration-separated products exhibited higher antioxidant activities as the MW cutoff increased (48–51, 53, 54). In addition, smaller peptides exhibited greater resistance to enzymatic degradation in the gastrointestinal tract and plasma, thus facilitating their absorption. Xu et al. (52) demonstrated that low-molecular-weight peptides were easily transported than the larger fraction in a Caco-2 cell absorption model.

Although the AOC potential of Alc-UF3P appeared more pronounced than that of Sav-UF3P, to the best of our knowledge, this study is among the few to report AOC activity in Savinase-digested tuna by-products. Savinase has been mainly used to produce hydrolysate from proteins of lentil, and fishes, including blue whiting, hake and eel (55). This finding is relevant from both biological and pharmaceutical perspectives, considering that ROS and free radicals exert a harmful effect on cellular and tissue homeostasis. Furthermore, food industry has also shown increasing interest in these bioactive molecules, as lipid oxidation remains a major concern for food deterioration (56). Our data suggested that low-MW Alcalase- and Savinase-derived hydrolysates could represent a potential natural alternative to synthetic antioxidants such as butylated hydroxytoluene (57–59), tert-butylhydroquinone (58, 60, 61), butylated hydroxyanisole (57, 58, 62), and propyl gallate (60, 63), commonly used in food industry, are subject to strict safety regulations (56). However, this application would require dedicated stability and food-matrix studies, which were beyond the scope of the present work.

The EC50 values calculated for the DPPH and ABTS assays (Table 2) further support the trend of increasing antioxidant activity with decreasing peptide molecular weight. Most notably, Alc-UF3P and Sav-UF3P displayed EC50 values below the lowest tested concentration, indicating that their antioxidant potency extends beyond the concentration range explored in this study and reinforcing, from an independent statistical parameter, the marked antioxidant activity already highlighted in section 3.2.

Beyond AOC, we also investigated the ACEi effects of Alcalase and Savinase peptides fractions. Indeed, low-MW peptides resulting from collagen digestion showed enhanced ACEi activity than the larger fractions (43). Our results showed that Alcalase digestion had higher ACEi effects than Savinase hydrolysates, possibly reflecting the different cleavage specificity of the two enzymes. Furthermore, as for the AOC, an inverse association between MW and ACEi potential emerged. In particular, Alc-UF3P and Sav-UF3P showed the higher ACEi effect for all the concentration tested. These findings are in line with literature data reporting that large sized peptides have shown more difficulties in entering and binding the active site of ACE compared to the smaller ones (64). Indeed, low MW peptide fractions obtained by protein hydrolysates from seafood, including tuna, evidenced highest ACEi activities (65–70). Analogously, Zheng et al. (69) compared Alcalase digestion with five other proteases to obtain bioactive hydrolysates from Skipjack tuna muscle. Their data revealed that Alcalase digestion was the most effective, yielding hydrolysates with the highest ACEi activity. It should be noted that, in the absence of a reference ACE inhibitor, such as captopril, tested under the same experimental conditions, the comparison between the inhibitory activity of our peptide fractions and clinically used ACE inhibitors remains indirect. Finally, we have to point out that, as observed by the AOC, our data revealed an appreciable ACEi effect from Sav-digested tuna by-products, although Alcalase showed stronger effects. To the best of our knowledge, we are among the few that reported ACEi activity in tuna by-products due to Sav digestion and ultrafiltration, although this remains to be validated *in vivo*. Evidence of ACEi peptide recovery is documented in both vegetable and fish byproducts (71, 72). Garcia-Mora et al. (71) reported that Alcalase and Savinase digestion of lentil protein concentrates produces hydrolysates with an appreciable ACEi activity. Analogously, Krichen et al. (72) used Savinase to obtain low-MW peptides with ACEi effect from shrimp waste.

The promising AOC and ACEi effects of the Alc-UF3P and Sav-UF3P evidenced by our data encouraged us to identify their aminoacidic composition, to provide further evidence supporting the higher bioactive potential of these fractions compared to others and to the crude hydrolysates. Indeed, the nature and proportion of hydrophobic amino acids such as alanine, phenylalanine, proline, valine, leucine, and isoleucine determine the efficacy and bioactivity of the peptides (73, 74). Looking at our data, the peptide fraction Alc-UF3P exhibited a distinct amino acid composition compared to the Sav-UF3P fraction, characterized by significantly higher levels of acidic residues (Asp and Glu), proline, and glycine, consistent with reports that Asp/Glu contribute to radical scavenging (75–78) and that Pro-rich peptides are associated with antihypertensive activity (79–81) These findings suggest that the aminoacidic composition of Alc-UF3P may contribute to its enhanced bioactivity, supporting its potential as a candidate for future nutraceutical or therapeutic investigation, pending peptide sequence and functional validation. Although the aminoacidic composition alone cannot establish a direct link with bioactivity, our results pave the way for tailored analysis addressing peptide sequence, structure, and peptide-enzyme interactions.

On the other hand, when considering the abundance of essential amino acids (EEAs), the Sav-UF3P exhibited statistically significant higher values compared to Alc-UF3P fraction. This is consistent with reports assuming that enzymatic hydrolysis can enhance the content of EEAs in protein hydrolysates (82). This suggests that the Sav-derived low-MW fraction could hold nutritional interest for future nutraceutical applications, warranting further investigation.

Finally, we evaluated the cytotoxicity of Alc-UF3P and Sav-UF3P fractions to confirm the absence of detectable cytotoxic effects that could limit their potential for future peptide-based applications. Our results demonstrated that no cytotoxic effects were observed in any of the tested cell lines, at any concentration, up to 72 h of exposure. Considering that the cytotoxicity assessment using a single cell line may not provide a complete understanding of substance’s biological effects, we used either intestinal (CCD-841-CON and Caco-2) and dermal (HDFa) cell lines to test the biological effect of Alc-UF3P and Sav-3UFP. Indeed, diverse cell lines respond differently to the same compound because of variation in metabolism, genetic background and receptor expression, thus resulting in different cytotoxic effects (83, 84). Additionally, the use of different concentrations has been properly chosen to verify any hormetic effect, limiting or masking the cytotoxicity of the peptide fractions. This approach strengthens the relevance of our findings, as the evaluation of viable cells following exposure to natural substances is a key step in many applications ranging from toxicological safety testing to drug discovery (84). Moreover, the inclusion of both intestinal and dermal cell lines offers insights that could support further studies investigating for oral and topical delivery routes.

Nevertheless, this study has some limitations. Most notably, the lack of peptide sequencing for Alc-UF3P and Sav-UF3P fractions, as well as the absence of a reference inhibitor, such as captopril, in the ACE inhibitory assay, which limits direct comparison with pharmaceutical standards. Related to this, our characterization of the active fractions relied on ultrafiltration cut-offs and overall amino acid composition, without determining the actual molecular weight distribution of the peptides within each fraction or identifying individual peptide sequences. As a result, the biological activities reported here reflect the behavior of peptide mixtures rather than of defined bioactive peptides, which may limit the mechanistic insight that can be drawn from our findings. Accordingly, the associations we propose between amino acid composition and antioxidant, or ACE-inhibitory activity should be regarded as plausible, literature-supported hypotheses rather than established causal relationships, since peptide sequence, structure, and peptide-enzyme interactions are ultimately the main determinants of bioactivity.

In conclusion, our data allowed us to demonstrate that: (i) we developed a sustainable-oriented and efficient model combining enzymatic digestion and ultrafiltration to obtain bioactive peptides from tuna by-products; (ii) we identified Alc-UF3P and Sav-UF3P fractions as those with the more prominent AOC and ACEi activities, without cytotoxic effect *in vitro*; and (iii) we demonstrated that Alc-UF3P exhibited the highest bioactive potential while Sav-UF3P showed greater nutritional value due to its EAAs content.

## 5 Conclusion

The results of this study demonstrate that enzymatic hydrolysis combined with membrane ultrafiltration is an effective and sustainable-oriented approach for recovering bioactive peptide fractions from tuna by-products. Notably, the Alcalase low-MW peptide fraction exhibited the most promising in vitro antioxidant and ACEi activities. To the best of our knowledge, this study is among the few reporting AOC activity in Savinase digested tuna by-products. Our results demonstrated that Savinase low-MW peptide fraction contained higher concentration of EEAs. Overall, we developed a process combining enzymatic hydrolysis at low temperature and ultrafiltration procedures for the valorization of tuna by-products, an ongoing scientific and technological challenge. The health-promoting and commercial potential of these peptide fractions remains to be established, as it depends on future digestion, bioavailability, and *in vivo* studies, as well as on the significant technological and regulatory steps needed before any industrial or nutraceutical application can be considered.

## Data Availability

The original contributions presented in the study are included in the article/Supplementary material, further inquiries can be directed to the corresponding authors.
