# Supplementary material for: Sustainability-oriented recovery of antioxidant and ACE-inhibitory low-molecular-weight peptide fractions from Thunnus albacares by-products via enzymatic hydrolysis and membrane ultrafiltration
Source: Front Nutr. 2026 Aug 21;13:1813218. doi: 10.3389/fnut.2026.1813218 (PMC13541559; doi:10.3389/fnut.2026.1813218)
Supplement: Supplementary file 1 [file Table_1.DOCX]

Supplementary Material

# 1 Supplementary Tables

Table S1. LC-MS/MS instrumental conditions for amino acid composition of bioactive peptide fractions

| **HPLC conditions** | |
| --- | --- |
|  | |
| **Stationary phase**: Kinetex Polar C18 3 × 100 mm, 2.6 µm particle size | |
| **Mobile phase:** A: Water containing 0.01% formic acid  B: Acetonitrile  Time program: B conc.14 % (0-3 min) -100%(10min) - 14%(10.01-15min) | |
| **Flow rate:** 0.6 mL/min | |
| **Injection volume:** 15 uL | |
| **Column temperature:** 40 °C | |
|  | |
| **Mass conditions** | |
|  | |
| **Ionization:** ESI, Positive MRM mode | |
| **MRM Transition** | |
| Trp | 205.10>188.10 |
| Phe | 166.10>120.10 |
| Tyr | 182.10>136.00 |
| Met | 150.10>56.10 |
| Lue, Lle | 132.10>86.15 |
| Val | 118.10>72.05 |
| Glu | 148.10>84.10 |
| Pro | 116.10>70.10 |
| Asp | 134.20>74.10 |
| Thr | 120.10>74.00 |
| Ala | 90.10>44.10 |
| Ser | 106.10>60.20 |
| Gln | 147.10>84.10 |
| Gly | 76.20>29.90 |
| Asn | 133.10>74.05 |
| (Cys)2 | 241.00>151.95 |
| His | 156.10>110.10 |
| Lys | 147.10>84.10 |
| Arg | 175.10>70.10 |

**Table S2** - Statistical analysis of DPPH assay results for Alcalase total hydrolysate (Alc-H) and its peptide fractions of varying molecular weights (Alc-UF10R, Alc-UF5R, Alc-UF3R, Alc-UF3P), performed using two-way ANOVA followed by Tukey’s multiple comparison test.

| Table Analyzed | Alcalase DPPH activity of hydrolysate and peptide fractions |
| --- | --- |

| Two-way ANOVA | Ordinary |
| --- | --- |
| Alpha | 0,05 |

| Source of Variation | % of total  variation | P value | P value  summary | Significant? |  |
| --- | --- | --- | --- | --- | --- |
| Interaction | 6,009 | <0.0001 | **** | Yes |  |
| Row Factor | 55,87 | <0.0001 | **** | Yes |  |
| Column Factor | 34,59 | <0.0001 | **** | Yes |  |
|  |  |  |  |  |  |
| ANOVA table | SS | DF | MS | F (DFn, DFd) | P value |
| Interaction | 49771 | 12 | 4148 | F (12, 100) = 14.19 | P<0.0001 |
| Row Factor | 462796 | 4 | 115699 | F (4, 100) = 395.8 | P<0.0001 |
| Column Factor | 286528 | 3 | 95509 | F (3, 100) = 326.7 | P<0.0001 |
| Residual | 29235 | 100 | 292,4 |  |  |
|  |  |  |  |  |  |
| Data summary |  |  |  |  |  |
| Number of columns (Column Factor) | 4 |  |  |  |  |
| Number of rows (Row Factor) | 5 |  |  |  |  |
| Number of values | 120 |  |  |  |  |

Multiple comparison

Within each column, compare rows (simple effects within columns)

| Number of families | 4 |
| --- | --- |
| Number of comparisons per family | 10 |
| Alpha | 0,05 |

| Tukey's multiple comparisons test | Mean Diff, | 95,00% CI of diff, | Below threshold? | Summary | Adjusted P Value |
| --- | --- | --- | --- | --- | --- |
|  |  |  |  |  |  |
| 100 μg/ml |  |  |  |  |  |
| Alc-H vs. Alc-UF10R | 142,3 | 114,9 to 169,7 | Yes | **** | <0,0001 |
| Alc-H vs. Alc-UF5R | 65,83 | 38,40 to 93,25 | Yes | **** | <0,0001 |
| Alc-H vs. Alc-UF3R | 28,23 | 0,8093 to 55,66 | Yes | * | 0,0403 |
| Alc-H vs. Alc-UF3P | -49,04 | -76,46 to -21,61 | Yes | **** | <0,0001 |
| Alc-UF10R vs. Alc-UF5R | -76,49 | -103,9 to -49,06 | Yes | **** | <0,0001 |
| Alc-UF10R vs. Alc-UF3R | -114,1 | -141,5 to -86,66 | Yes | **** | <0,0001 |
| Alc-UF10R vs. Alc-UF3P | -191,4 | -218,8 to -163,9 | Yes | **** | <0,0001 |
| Alc-UF5R vs. Alc-UF3R | -37,59 | -65,02 to -10,17 | Yes | ** | 0,0022 |
| Alc-UF5R vs. Alc-UF3P | -114,9 | -142,3 to -87,44 | Yes | **** | <0,0001 |
| Alc-UF3R vs. Alc-UF3P | -77,27 | -104,7 to -49,85 | Yes | **** | <0,0001 |
|  |  |  |  |  |  |
| 50 μg/ml |  |  |  |  |  |
| Alc-H vs. Alc-UF10R | 130,1 | 102,6 to 157,5 | Yes | **** | <0,0001 |
| Alc-H vs. Alc-UF5R | 62,91 | 35,49 to 90,34 | Yes | **** | <0,0001 |
| Alc-H vs. Alc-UF3R | -2,42 | -29,85 to 25,01 | No | ns | 0,9992 |
| Alc-H vs. Alc-UF3P | -62,91 | -90,34 to -35,49 | Yes | **** | <0,0001 |
| Alc-UF10R vs. Alc-UF5R | -67,15 | -94,57 to -39,72 | Yes | **** | <0,0001 |
| Alc-UF10R vs. Alc-UF3R | -132,5 | -159,9 to -105,1 | Yes | **** | <0,0001 |
| Alc-UF10R vs. Alc-UF3P | -193 | -220,4 to -165,6 | Yes | **** | <0,0001 |
| Alc-UF5R vs. Alc-UF3R | -65,33 | -92,76 to -37,91 | Yes | **** | <0,0001 |
| Alc-UF5R vs. Alc-UF3P | -125,8 | -153,3 to -98,40 | Yes | **** | <0,0001 |
| Alc-UF3R vs. Alc-UF3P | -60,49 | -87,92 to -33,07 | Yes | **** | <0,0001 |
|  |  |  |  |  |  |
| 25 μg/ml |  |  |  |  |  |
| Alc-H vs. Alc-UF10R | 72,37 | 44,95 to 99,80 | Yes | **** | <0,0001 |
| Alc-H vs. Alc-UF5R | 11,62 | -15,80 to 39,05 | No | ns | 0,7643 |
| Alc-H vs. Alc-UF3R | -55,97 | -83,40 to -28,55 | Yes | **** | <0,0001 |
| Alc-H vs. Alc-UF3P | -101,1 | -128,6 to -73,71 | Yes | **** | <0,0001 |
| Alc-UF10R vs. Alc-UF5R | -60,75 | -88,18 to -33,33 | Yes | **** | <0,0001 |
| Alc-UF10R vs. Alc-UF3R | -128,3 | -155,8 to -100,9 | Yes | **** | <0,0001 |
| Alc-UF10R vs. Alc-UF3P | -173,5 | -200,9 to -146,1 | Yes | **** | <0,0001 |
| Alc-UF5R vs. Alc-UF3R | -67,59 | -95,02 to -40,17 | Yes | **** | <0,0001 |
| Alc-UF5R vs. Alc-UF3P | -112,8 | -140,2 to -85,33 | Yes | **** | <0,0001 |
| Alc-UF3R vs. Alc-UF3P | -45,17 | -72,59 to -17,74 | Yes | *** | 0,0001 |
|  |  |  |  |  |  |
| 12.5 μg/ml |  |  |  |  |  |
| Alc-H vs. Alc-UF10R | 21,16 | -6,263 to 48,59 | No | ns | 0,2101 |
| Alc-H vs. Alc-UF5R | -58,54 | -85,96 to -31,11 | Yes | **** | <0,0001 |
| Alc-H vs. Alc-UF3R | -92,09 | -119,5 to -64,67 | Yes | **** | <0,0001 |
| Alc-H vs. Alc-UF3P | -179,2 | -206,6 to -151,8 | Yes | **** | <0,0001 |
| Alc-UF10R vs. Alc-UF5R | -79,7 | -107,1 to -52,28 | Yes | **** | <0,0001 |
| Alc-UF10R vs. Alc-UF3R | -113,3 | -140,7 to -85,83 | Yes | **** | <0,0001 |
| Alc-UF10R vs. Alc-UF3P | -200,4 | -227,8 to -172,9 | Yes | **** | <0,0001 |
| Alc-UF5R vs. Alc-UF3R | -33,56 | -60,98 to -6,130 | Yes | ** | 0,0084 |
| Alc-UF5R vs. Alc-UF3P | -120,7 | -148,1 to -93,24 | Yes | **** | <0,0001 |
| Alc-UF3R vs. Alc-UF3P | -87,11 | -114,5 to -59,68 | Yes | **** | <0,0001 |

**Table S3** – Statistical analysis of DPPH assay results for Savinase total hydrolysate (Sav-H) and its peptide fractions of varying molecular weights (Sav-UF10R, Sav-UF5R, Sav-UF3R, Sav-UF3P), performed using two-way ANOVA followed by Tukey’s multiple comparison test

| Table Analyzed | Savinase DPPH activity of hydrolysate and peptide fractions |
| --- | --- |

| Two-way ANOVA | Ordinary |
| --- | --- |
| Alpha | 0,05 |

| Source of Variation | % of total variation | P value | P value summary | Significant? |  |
| --- | --- | --- | --- | --- | --- |
| Interaction | 2,872 | <0.0001 | **** | Yes |  |
| Row Factor | 71,46 | <0.0001 | **** | Yes |  |
| Column Factor | 21,9 | <0.0001 | **** | Yes |  |
|  |  |  |  |  |  |
| ANOVA table | SS | DF | MS | F (DFn, DFd) | P value |
| Interaction | 19458 | 12 | 1622 | F (12, 100) = 6.347 | P<0.0001 |
| Row Factor | 484159 | 4 | 121040 | F (4, 100) = 473.8 | P<0.0001 |
| Column Factor | 148394 | 3 | 49465 | F (3, 100) = 193.6 | P<0.0001 |
| Residual | 25547 | 100 | 255,5 |  |  |
|  |  |  |  |  |  |
| Data summary |  |  |  |  |  |
| Number of columns (Column Factor) | 4 |  |  |  |  |
| Number of rows (Row Factor) | 5 |  |  |  |  |
| Number of values | 120 |  |  |  |  |

Multiple comparison

Within each column, compare rows (simple effects within columns)

| Number of families | 4 |
| --- | --- |
| Number of comparisons per fam | 10 |
| Alpha | 0,05 |

| Tukey's multiple comparisons test | Mean Diff. | 95.00% CI of diff. | Below threshold? | Summary | Adjusted P Value |
| --- | --- | --- | --- | --- | --- |
|  |  |  |  |  |  |
| 100 µg/ml |  |  |  |  |  |
| Sav-H vs. Sav-UF10R | 75,56 | 49.92 to 101.2 | Yes | **** | <0.0001 |
| Sav-H vs. Sav-UF5R | 18,02 | -7.613 to 43.66 | No | ns | 0.2966 |
| Sav-H vs. Sav-UF3R | -46,79 | -72.43 to -21.15 | Yes | **** | <0.0001 |
| Sav-H vs. Sav-UF3P | -115,9 | -141.6 to -90.29 | Yes | **** | <0.0001 |
| Sav-UF10R vs. Sav-UF5R | -57,53 | -83.17 to -31.89 | Yes | **** | <0.0001 |
| Sav-UF10R vs. Sav-UF3R | -122,3 | -148.0 to -96.71 | Yes | **** | <0.0001 |
| Sav-UF10R vs. Sav-UF3P | -191,5 | -217.1 to -165.8 | Yes | **** | <0.0001 |
| Sav-UF5R vs. Sav-UF3R | -64,81 | -90.45 to -39.18 | Yes | **** | <0.0001 |
| Sav-UF5R vs. Sav-UF3P | -134 | -159.6 to -108.3 | Yes | **** | <0.0001 |
| Sav-UF3R vs. Sav-UF3P | -69,14 | -94.77 to -43.50 | Yes | **** | <0.0001 |
|  |  |  |  |  |  |
| 50 µg/ml |  |  |  |  |  |
| Sav-H vs. Sav-UF10R | 49,63 | 23.99 to 75.27 | Yes | **** | <0.0001 |
| Sav-H vs. Sav-UF5R | -15,19 | -40.82 to 10.45 | No | ns | 0.4723 |
| Sav-H vs. Sav-UF3R | -88,64 | -114.3 to -63.00 | Yes | **** | <0.0001 |
| Sav-H vs. Sav-UF3P | -136,2 | -161.8 to -110.5 | Yes | **** | <0.0001 |
| Sav-UF10R vs. Sav-UF5R | -64,81 | -90.45 to -39.18 | Yes | **** | <0.0001 |
| Sav-UF10R vs. Sav-UF3R | -138,3 | -163.9 to -112.6 | Yes | **** | <0.0001 |
| Sav-UF10R vs. Sav-UF3P | -185,8 | -211.4 to -160.2 | Yes | **** | <0.0001 |
| Sav-UF5R vs. Sav-UF3R | -73,46 | -99.09 to -47.82 | Yes | **** | <0.0001 |
| Sav-UF5R vs. Sav-UF3P | -121 | -146.6 to -95.35 | Yes | **** | <0.0001 |
| Sav-UF3R vs. Sav-UF3P | -47,53 | -73.17 to -21.89 | Yes | **** | <0.0001 |
|  |  |  |  |  |  |
| 25 µg/ml |  |  |  |  |  |
| Sav-H vs. Sav-UF10R | 7,161 | -18.48 to 32.80 | No | ns | 0.937 |
| Sav-H vs. Sav-UF5R | -56,79 | -82.43 to -31.15 | Yes | **** | <0.0001 |
| Sav-H vs. Sav-UF3R | -130,2 | -155.9 to -104.6 | Yes | **** | <0.0001 |
| Sav-H vs. Sav-UF3P | -164,8 | -190.5 to -139.2 | Yes | **** | <0.0001 |
| Sav-UF10R vs. Sav-UF5R | -63,95 | -89.59 to -38.31 | Yes | **** | <0.0001 |
| Sav-UF10R vs. Sav-UF3R | -137,4 | -163.0 to -111.8 | Yes | **** | <0.0001 |
| Sav-UF10R vs. Sav-UF3P | -172 | -197.6 to -146.3 | Yes | **** | <0.0001 |
| Sav-UF5R vs. Sav-UF3R | -73,46 | -99.09 to -47.82 | Yes | **** | <0.0001 |
| Sav-UF5R vs. Sav-UF3P | -108 | -133.7 to -82.39 | Yes | **** | <0.0001 |
| Sav-UF3R vs. Sav-UF3P | -34,57 | -60.21 to -8.931 | Yes | ** | 0.0027 |
|  |  |  |  |  |  |
| 12.5 µg/ml |  |  |  |  |  |
| Sav-H vs. Sav-UF10R | 8,827 | -16.81 to 34.46 | No | ns | 0.8737 |
| Sav-H vs. Sav-UF5R | -46,79 | -72.43 to -21.15 | Yes | **** | <0.0001 |
| Sav-H vs. Sav-UF3R | -118,6 | -144.3 to -93.00 | Yes | **** | <0.0001 |
| Sav-H vs. Sav-UF3P | -136,2 | -161.8 to -110.5 | Yes | **** | <0.0001 |
| Sav-UF10R vs. Sav-UF5R | -55,62 | -81.25 to -29.98 | Yes | **** | <0.0001 |
| Sav-UF10R vs. Sav-UF3R | -127,5 | -153.1 to -101.8 | Yes | **** | <0.0001 |
| Sav-UF10R vs. Sav-UF3P | -145 | -170.6 to -119.4 | Yes | **** | <0.0001 |
| Sav-UF5R vs. Sav-UF3R | -71,85 | -97.49 to -46.21 | Yes | **** | <0.0001 |
| Sav-UF5R vs. Sav-UF3P | -89,38 | -115.0 to -63.75 | Yes | **** | <0.0001 |
| Sav-UF3R vs. Sav-UF3P | -17,53 | -43.17 to 8.106 | No | ns | 0.3243 |

**Table S4** – Statistical analysis of ABTS assay results for Alcalase total hydrolysate (Alc-H) and its peptide fractions of varying molecular weights (Alc-UF10R, Alc-UF5R, Alc-UF3R, Alc-UF3P), performed using two-way ANOVA followed by Tukey’s multiple comparison test.

| Table Analyzed | Alcalase ABTS activity of hydrolysate and peptide fractions |
| --- | --- |

| Two-way ANOVA | Ordinary |
| --- | --- |
| Alpha | 0,05 |

| Source of Variation | % of total variation | P value | P value summary | Significant? |  |
| --- | --- | --- | --- | --- | --- |
| Interaction | 4,257 | <0.0001 | **** | Yes |  |
| Row Factor | 72,07 | <0.0001 | **** | Yes |  |
| Column Factor | 22,54 | <0.0001 | **** | Yes |  |
|  |  |  |  |  |  |
| ANOVA table | SS | DF | MS | F (DFn, DFd) | P value |
| Interaction | 2635 | 12 | 219,6 | F (12, 100) = 31.25 | P<0.0001 |
| Row Factor | 44618 | 4 | 11154 | F (4, 100) = 1587 | P<0.0001 |
| Column Factor | 13954 | 3 | 4651 | F (3, 100) = 661.9 | P<0.0001 |
| Residual | 702.8 | 100 | 7,028 |  |  |
|  |  |  |  |  |  |
| Data summary |  |  |  |  |  |
| Number of columns (Column Factor) | 4 |  |  |  |  |
| Number of rows (Row Factor) | 5 |  |  |  |  |
| Number of values | 120 |  |  |  |  |

Multiple comparison

Within each column, compare rows (simple effects within columns)

| Number of families | 4 |
| --- | --- |
| Number of comparisons per family | 10 |
| Alpha | 0.05 |

| Tukey's multiple comparisons test | Mean Diff. | 95.00% CI of diff. | Below threshold? | Summary | Adjusted P Value |
| --- | --- | --- | --- | --- | --- |
|  |  |  |  |  |  |
| 100 µg/ml |  |  |  |  |  |
| Alc-H vs. Alc-UF10R | 33,54 | 29.29 to 37.79 | Yes | **** | <0.0001 |
| Alc-H vs. Alc-UF5R | 46 | 41.75 to 50.25 | Yes | **** | <0.0001 |
| Alc-H vs. Alc-UF3R | 11 | 6.748 to 15.25 | Yes | **** | <0.0001 |
| Alc-H vs. Alc-UF3P | -6 | -10.25 to -1.748 | Yes | ** | 0.0015 |
| Alc-UF10R vs. Alc-UF5R | 12,46 | 8.208 to 16.71 | Yes | **** | <0.0001 |
| Alc-UF10R vs. Alc-UF3R | -22,54 | -26.79 to -18.29 | Yes | **** | <0.0001 |
| Alc-UF10R vs. Alc-UF3P | -39,54 | -43.79 to -35.29 | Yes | **** | <0.0001 |
| Alc-UF5R vs. Alc-UF3R | -35 | -39.25 to -30.75 | Yes | **** | <0.0001 |
| Alc-UF5R vs. Alc-UF3P | -52 | -56.25 to -47.75 | Yes | **** | <0.0001 |
| Alc-UF3R vs. Alc-UF3P | -17 | -21.25 to -12.75 | Yes | **** | <0.0001 |
|  |  |  |  |  |  |
| 50 µg/ml |  |  |  |  |  |
| Alc-H vs. Alc-UF10R | 26,87 | 22.62 to 31.12 | Yes | **** | <0.0001 |
| Alc-H vs. Alc-UF5R | 33 | 28.75 to 37.25 | Yes | **** | <0.0001 |
| Alc-H vs. Alc-UF3R | -0,5 | -4.752 to 3.752 | No | ns | 0.9975 |
| Alc-H vs. Alc-UF3P | -13 | -17.25 to -8.748 | Yes | **** | <0.0001 |
| Alc-UF10R vs. Alc-UF5R | 6,13 | 1.878 to 10.38 | Yes | ** | 0.0011 |
| Alc-UF10R vs. Alc-UF3R | -27,37 | -31.62 to -23.12 | Yes | **** | <0.0001 |
| Alc-UF10R vs. Alc-UF3P | -39,87 | -44.12 to -35.62 | Yes | **** | <0.0001 |
| Alc-UF5R vs. Alc-UF3R | -33,5 | -37.75 to -29.25 | Yes | **** | <0.0001 |
| Alc-UF5R vs. Alc-UF3P | -46 | -50.25 to -41.75 | Yes | **** | <0.0001 |
| Alc-UF3R vs. Alc-UF3P | -12,5 | -16.75 to -8.248 | Yes | **** | <0.0001 |
|  |  |  |  |  |  |
| 25 µg/ml |  |  |  |  |  |
| Alc-H vs. Alc-UF10R | 28 | 23.75 to 32.25 | Yes | **** | <0.0001 |
| Alc-H vs. Alc-UF5R | 36 | 31.75 to 40.25 | Yes | **** | <0.0001 |
| Alc-H vs. Alc-UF3R | -5 | -9.252 to -0.7479 | Yes | * | 0.0127 |
| Alc-H vs. Alc-UF3P | -19 | -23.25 to -14.75 | Yes | **** | <0.0001 |
| Alc-UF10R vs. Alc-UF5R | 8 | 3.748 to 12.25 | Yes | **** | <0.0001 |
| Alc-UF10R vs. Alc-UF3R | -33 | -37.25 to -28.75 | Yes | **** | <0.0001 |
| Alc-UF10R vs. Alc-UF3P | -47 | -51.25 to -42.75 | Yes | **** | <0.0001 |
| Alc-UF5R vs. Alc-UF3R | -41 | -45.25 to -36.75 | Yes | **** | <0.0001 |
| Alc-UF5R vs. Alc-UF3P | -55 | -59.25 to -50.75 | Yes | **** | <0.0001 |
| Alc-UF3R vs. Alc-UF3P | -14 | -18.25 to -9.748 | Yes | **** | <0.0001 |
|  |  |  |  |  |  |
| 12.5 µg/ml |  |  |  |  |  |
| Alc-H vs. Alc-UF10R | 11 | 6.748 to 15.25 | Yes | **** | <0.0001 |
| Alc-H vs. Alc-UF5R | 20 | 15.75 to 24.25 | Yes | **** | <0.0001 |
| Alc-H vs. Alc-UF3R | -9 | -13.25 to -4.748 | Yes | **** | <0.0001 |
| Alc-H vs. Alc-UF3P | -41 | -45.25 to -36.75 | Yes | **** | <0.0001 |
| Alc-UF10R vs. Alc-UF5R | 9 | 4.748 to 13.25 | Yes | **** | <0.0001 |
| Alc-UF10R vs. Alc-UF3R | -20 | -24.25 to -15.75 | Yes | **** | <0.0001 |
| Alc-UF10R vs. Alc-UF3P | -52 | -56.25 to -47.75 | Yes | **** | <0.0001 |
| Alc-UF5R vs. Alc-UF3R | -29 | -33.25 to -24.75 | Yes | **** | <0.0001 |
| Alc-UF5R vs. Alc-UF3P | -61 | -65.25 to -56.75 | Yes | **** | <0.0001 |
| Alc-UF3R vs. Alc-UF3P | -32 | -36.25 to -27.75 | Yes | **** | <0.0001 |

**Table S5** – Statistical analysis of ABTS assay results for Savinase total hydrolysate (Sav-H) and its peptide fractions of varying molecular weights (Sav-UF10R, Sav-UF5R, Sav-UF3R, Sav-UF3P), performed using two-way ANOVA followed by Tukey’s multiple comparison test.

| Table Analyzed | Savinase ABTS activity of hydrolysate and peptide fractions |
| --- | --- |

| Two-way ANOVA | Ordinary |
| --- | --- |
| Alpha | 0,05 |

| Source of Variation | % of total variation | P value | P value summary | Significant? |  |
| --- | --- | --- | --- | --- | --- |
| Interaction | 6,155 | <0.0001 | **** | Yes |  |
| Row Factor | 65,8 | <0.0001 | **** | Yes |  |
| Column Factor | 26,07 | <0.0001 | **** | Yes |  |
|  |  |  |  |  |  |
| ANOVA table | SS | DF | MS | F (DFn, DFd) | P value |
| Interaction | 2341 | 12 | 195,1 | F (12, 100) = 25.97 | P<0.0001 |
| Row Factor | 25028 | 4 | 6257 | F (4, 100) = 832.9 | P<0.0001 |
| Column Factor | 9917 | 3 | 3306 | F (3, 100) = 440.0 | P<0.0001 |
| Residual | 751,3 | 100 | 7,513 |  |  |
|  |  |  |  |  |  |
| Data summary |  |  |  |  |  |
| Number of columns (Column Factor) | 4 |  |  |  |  |
| Number of rows (Row Factor) | 5 |  |  |  |  |
| Number of values | 120 |  |  |  |  |

Multiple comparison

Within each column, compare rows (simple effects within columns)

| Number of families | 4 |
| --- | --- |
| Number of comparisons per family | 10 |
| Alpha | 0,05 |

| Tukey's multiple comparisons test | Mean Diff. | 95.00% CI of diff. | Below threshold? | Summary | Adjusted P Value |
| --- | --- | --- | --- | --- | --- |
|  |  |  |  |  |  |
| 100 µg/ml |  |  |  |  |  |
| Sav-H vs. Sav-UF10R | 36 | 31.60 to 40.40 | Yes | **** | <0.0001 |
| Sav-H vs. Sav-UF5R | 25 | 20.60 to 29.40 | Yes | **** | <0.0001 |
| Sav-H vs. Sav-UF3R | 10 | 5.604 to 14.40 | Yes | **** | <0.0001 |
| Sav-H vs. Sav-UF3P | -6 | -10.40 to -1.604 | Yes | ** | 0.0023 |
| Sav-UF10R vs. Sav-UF5R | -11 | -15.40 to -6.604 | Yes | **** | <0.0001 |
| Sav-UF10R vs. Sav-UF3R | -26 | -30.40 to -21.60 | Yes | **** | <0.0001 |
| Sav-UF10R vs. Sav-UF3P | -42 | -46.40 to -37.60 | Yes | **** | <0.0001 |
| Sav-UF5R vs. Sav-UF3R | -15 | -19.40 to -10.60 | Yes | **** | <0.0001 |
| Sav-UF5R vs. Sav-UF3P | -31 | -35.40 to -26.60 | Yes | **** | <0.0001 |
| Sav-UF3R vs. Sav-UF3P | -16 | -20.40 to -11.60 | Yes | **** | <0.0001 |
|  |  |  |  |  |  |
| 50 µg/ml |  |  |  |  |  |
| Sav-H vs. Sav-UF10R | 30 | 25.60 to 34.40 | Yes | **** | <0.0001 |
| Sav-H vs. Sav-UF5R | 15 | 10.60 to 19.40 | Yes | **** | <0.0001 |
| Sav-H vs. Sav-UF3R | -2 | -6.396 to 2.396 | No | ns | 0.7138 |
| Sav-H vs. Sav-UF3P | -13 | -17.40 to -8.604 | Yes | **** | <0.0001 |
| Sav-UF10R vs. Sav-UF5R | -15 | -19.40 to -10.60 | Yes | **** | <0.0001 |
| Sav-UF10R vs. Sav-UF3R | -32 | -36.40 to -27.60 | Yes | **** | <0.0001 |
| Sav-UF10R vs. Sav-UF3P | -43 | -47.40 to -38.60 | Yes | **** | <0.0001 |
| Sav-UF5R vs. Sav-UF3R | -17 | -21.40 to -12.60 | Yes | **** | <0.0001 |
| Sav-UF5R vs. Sav-UF3P | -28 | -32.40 to -23.60 | Yes | **** | <0.0001 |
| Sav-UF3R vs. Sav-UF3P | -11 | -15.40 to -6.604 | Yes | **** | <0.0001 |
|  |  |  |  |  |  |
| 25 µg/ml |  |  |  |  |  |
| Sav-H vs. Sav-UF10R | 24,8 | 20.40 to 29.20 | Yes | **** | <0.0001 |
| Sav-H vs. Sav-UF5R | 10 | 5.604 to 14.40 | Yes | **** | <0.0001 |
| Sav-H vs. Sav-UF3R | -7 | -11.40 to -2.604 | Yes | *** | 0.0002 |
| Sav-H vs. Sav-UF3P | -15 | -19.40 to -10.60 | Yes | **** | <0.0001 |
| Sav-UF10R vs. Sav-UF5R | -14,8 | -19.20 to -10.40 | Yes | **** | <0.0001 |
| Sav-UF10R vs. Sav-UF3R | -31,8 | -36.20 to -27.40 | Yes | **** | <0.0001 |
| Sav-UF10R vs. Sav-UF3P | -39,8 | -44.20 to -35.40 | Yes | **** | <0.0001 |
| Sav-UF5R vs. Sav-UF3R | -17 | -21.40 to -12.60 | Yes | **** | <0.0001 |
| Sav-UF5R vs. Sav-UF3P | -25 | -29.40 to -20.60 | Yes | **** | <0.0001 |
| Sav-UF3R vs. Sav-UF3P | -8 | -12.40 to -3.604 | Yes | **** | <0.0001 |
|  |  |  |  |  |  |
| 12.5 µg/ml |  |  |  |  |  |
| Sav-H vs. Sav-UF10R | 7,5 | 3.104 to 11.90 | Yes | **** | <0.0001 |
| Sav-H vs. Sav-UF5R | 0 | -4.396 to 4.396 | No | ns | >0.9999 |
| Sav-H vs. Sav-UF3R | -22 | -26.40 to -17.60 | Yes | **** | <0.0001 |
| Sav-H vs. Sav-UF3P | -35 | -39.40 to -30.60 | Yes | **** | <0.0001 |
| Sav-UF10R vs. Sav-UF5R | -7,5 | -11.90 to -3.104 | Yes | **** | <0.0001 |
| Sav-UF10R vs. Sav-UF3R | -29,5 | -33.90 to -25.10 | Yes | **** | <0.0001 |
| Sav-UF10R vs. Sav-UF3P | -42,5 | -46.90 to -38.10 | Yes | **** | <0.0001 |
| Sav-UF5R vs. Sav-UF3R | -22 | -26.40 to -17.60 | Yes | **** | <0.0001 |
| Sav-UF5R vs. Sav-UF3P | -35 | -39.40 to -30.60 | Yes | **** | <0.0001 |
| Sav-UF3R vs. Sav-UF3P | -13 | -17.40 to -8.604 | Yes | **** | <0.0001 |

**Table S6** – Statistical analysis of FRAP assay results for Alcalase total hydrolysate (Alc-H) and its peptide fractions of varying molecular weights (Alc-UF10R, Alc-UF5R, Alc-UF3R, Alc-UF3P), performed using two-way ANOVA followed by Tukey’s multiple comparison test

| Table Analyzed | Alcalase FRAP activity of hydrolysate and peptide fractions |
| --- | --- |

| Two-way ANOVA | Ordinary |
| --- | --- |
| Alpha | 0,05 |

| Source of Variation | % of total variation | P value | P value summary | Significant? |  |
| --- | --- | --- | --- | --- | --- |
| Interaction | 3,359 | <0.0001 | **** | Yes |  |
| Row Factor | 74,13 | <0.0001 | **** | Yes |  |
| Column Factor | 20,96 | <0.0001 | **** | Yes |  |
|  |  |  |  |  |  |
| ANOVA table | SS | DF | MS | F (DFn, DFd) | P value |
| Interaction | 2,524 | 12 | 0,2104 | F (12, 100) = 18.09 | P<0.0001 |
| Row Factor | 55,72 | 4 | 13,93 | F (4, 100) = 1198 | P<0.0001 |
| Column Factor | 15,75 | 3 | 5,251 | F (3, 100) = 451.5 | P<0.0001 |
| Residual | 1,163 | 100 | 0,01163 |  |  |
|  |  |  |  |  |  |
| Data summary |  |  |  |  |  |
| Number of columns (Column Factor) | 4 |  |  |  |  |
| Number of rows (Row Factor) | 5 |  |  |  |  |
| Number of values | 120 |  |  |  |  |

Multiple comparison

Within each column, compare rows (simple effects within columns)

| Number of families | 4 |
| --- | --- |
| Number of comparisons per family | 10 |
| Alpha | 0,05 |

| Tukey's multiple comparisons test | Mean Diff. | 95.00% CI of diff. | Below threshold? | Summary | Adjusted P Value |
| --- | --- | --- | --- | --- | --- |
|  |  |  |  |  |  |
| 100 µg/ml |  |  |  |  |  |
| Alc-H vs. Alc-UF10R | 1,2 | 1.027 to 1.373 | Yes | **** | <0.0001 |
| Alc-H vs. Alc-UF5R | 0,2 | 0.02702 to 0.3730 | Yes | * | 0.0149 |
| Alc-H vs. Alc-UF3R | -0,5 | -0.6730 to -0.3270 | Yes | **** | <0.0001 |
| Alc-H vs. Alc-UF3P | -0,83 | -1.003 to -0.6570 | Yes | **** | <0.0001 |
| Alc-UF10R vs. Alc-UF5R | -1 | -1.173 to -0.8270 | Yes | **** | <0.0001 |
| Alc-UF10R vs. Alc-UF3R | -1,7 | -1.873 to -1.527 | Yes | **** | <0.0001 |
| Alc-UF10R vs. Alc-UF3P | -2,03 | -2.203 to -1.857 | Yes | **** | <0.0001 |
| Alc-UF5R vs. Alc-UF3R | -0,7 | -0.8730 to -0.5270 | Yes | **** | <0.0001 |
| Alc-UF5R vs. Alc-UF3P | -1,03 | -1.203 to -0.8570 | Yes | **** | <0.0001 |
| Alc-UF3R vs. Alc-UF3P | -0,33 | -0.5030 to -0.1570 | Yes | **** | <0.0001 |
|  |  |  |  |  |  |
| 50 µg/ml |  |  |  |  |  |
| Alc-H vs. Alc-UF10R | 1,39 | 1.217 to 1.563 | Yes | **** | <0.0001 |
| Alc-H vs. Alc-UF5R | 0,27 | 0.09702 to 0.4430 | Yes | *** | 0.0003 |
| Alc-H vs. Alc-UF3R | -0,49 | -0.6630 to -0.3170 | Yes | **** | <0.0001 |
| Alc-H vs. Alc-UF3P | -0,83 | -1.003 to -0.6570 | Yes | **** | <0.0001 |
| Alc-UF10R vs. Alc-UF5R | -1,12 | -1.293 to -0.9470 | Yes | **** | <0.0001 |
| Alc-UF10R vs. Alc-UF3R | -1,88 | -2.053 to -1.707 | Yes | **** | <0.0001 |
| Alc-UF10R vs. Alc-UF3P | -2,22 | -2.393 to -2.047 | Yes | **** | <0.0001 |
| Alc-UF5R vs. Alc-UF3R | -0,76 | -0.9330 to -0.5870 | Yes | **** | <0.0001 |
| Alc-UF5R vs. Alc-UF3P | -1,1 | -1.273 to -0.9270 | Yes | **** | <0.0001 |
| Alc-UF3R vs. Alc-UF3P | -0,34 | -0.5130 to -0.1670 | Yes | **** | <0.0001 |
|  |  |  |  |  |  |
| 25 µg/ml |  |  |  |  |  |
| Alc-H vs. Alc-UF10R | 1,43 | 1.257 to 1.603 | Yes | **** | <0.0001 |
| Alc-H vs. Alc-UF5R | 0,61 | 0.4370 to 0.7830 | Yes | **** | <0.0001 |
| Alc-H vs. Alc-UF3R | -0,02 | -0.1930 to 0.1530 | No | ns | 0.9977 |
| Alc-H vs. Alc-UF3P | -0,64 | -0.8130 to -0.4670 | Yes | **** | <0.0001 |
| Alc-UF10R vs. Alc-UF5R | -0,82 | -0.9930 to -0.6470 | Yes | **** | <0.0001 |
| Alc-UF10R vs. Alc-UF3R | -1,45 | -1.623 to -1.277 | Yes | **** | <0.0001 |
| Alc-UF10R vs. Alc-UF3P | -2,07 | -2.243 to -1.897 | Yes | **** | <0.0001 |
| Alc-UF5R vs. Alc-UF3R | -0,63 | -0.8030 to -0.4570 | Yes | **** | <0.0001 |
| Alc-UF5R vs. Alc-UF3P | -1,25 | -1.423 to -1.077 | Yes | **** | <0.0001 |
| Alc-UF3R vs. Alc-UF3P | -0,62 | -0.7930 to -0.4470 | Yes | **** | <0.0001 |
|  |  |  |  |  |  |
| 12.5 µg/ml |  |  |  |  |  |
| Alc-H vs. Alc-UF10R | 0,45 | 0.2770 to 0.6230 | Yes | **** | <0.0001 |
| Alc-H vs. Alc-UF5R | 0 | -0.1730 to 0.1730 | No | ns | >0.9999 |
| Alc-H vs. Alc-UF3R | -0,89 | -1.063 to -0.7170 | Yes | **** | <0.0001 |
| Alc-H vs. Alc-UF3P | -1,22 | -1.393 to -1.047 | Yes | **** | <0.0001 |
| Alc-UF10R vs. Alc-UF5R | -0,45 | -0.6230 to -0.2770 | Yes | **** | <0.0001 |
| Alc-UF10R vs. Alc-UF3R | -1,34 | -1.513 to -1.167 | Yes | **** | <0.0001 |
| Alc-UF10R vs. Alc-UF3P | -1,67 | -1.843 to -1.497 | Yes | **** | <0.0001 |
| Alc-UF5R vs. Alc-UF3R | -0,89 | -1.063 to -0.7170 | Yes | **** | <0.0001 |
| Alc-UF5R vs. Alc-UF3P | -1,22 | -1.393 to -1.047 | Yes | **** | <0.0001 |
| Alc-UF3R vs. Alc-UF3P | -0,33 | -0.5030 to -0.1570 | Yes | **** | <0.0001 |

**Table S7** – Statistical analysis of FRAP assay results for Savinase total hydrolysate (Sav-H) and its peptide fractions of varying molecular weights (Sav-UF10R, Sav-UF5R, Sav-UF3R, Sav-UF3P), performed using two-way ANOVA followed by Tukey’s multiple comparison test

| Table Analyzed | Savinase FRAP activity of hydrolysate and peptide fractions |
| --- | --- |

| Two-way ANOVA | Ordinary |
| --- | --- |
| Alpha | 0,05 |

| Source of Variation | % of total variation | P value | P value summary | Significant? |  |
| --- | --- | --- | --- | --- | --- |
| Interaction | 3,585 | <0.0001 | **** | Yes |  |
| Row Factor | 67,02 | <0.0001 | **** | Yes |  |
| Column Factor | 27,91 | <0.0001 | **** | Yes |  |
|  |  |  |  |  |  |
| ANOVA table | SS | DF | MS | F (DFn, DFd) | P value |
| Interaction | 2,006 | 12 | 0,1672 | F (12, 100) = 19.99 | P<0.0001 |
| Row Factor | 37,51 | 4 | 9,377 | F (4, 100) = 1121 | P<0.0001 |
| Column Factor | 15,62 | 3 | 5,206 | F (3, 100) = 622.4 | P<0.0001 |
| Residual | 0,8365 | 100 | 0,008365 |  |  |
|  |  |  |  |  |  |
| Data summary |  |  |  |  |  |
| Number of columns (Column Factor) | 4 |  |  |  |  |
| Number of rows (Row Factor) | 5 |  |  |  |  |
| Number of values | 120 |  |  |  |  |

Multiple comparison

Within each column, compare rows (simple effects within columns)

| Number of families | 4 |
| --- | --- |
| Number of comparisons per family | 10 |
| Alpha | 0,05 |

| Tukey's multiple comparisons test | Mean Diff. | 95.00% CI of diff. | Below threshold? | Summary | Adjusted P Value |
| --- | --- | --- | --- | --- | --- |

| 100 µg/ml |  |  |  |  |  |
| --- | --- | --- | --- | --- | --- |
| Sav-H vs. Sav-UF10R | 1,27 | 1.123 to 1.417 | Yes | **** | <0.0001 |
| Sav-H vs. Sav-UF5R | 0,41 | 0.2633 to 0.5567 | Yes | **** | <0.0001 |
| Sav-H vs. Sav-UF3R | -0,3 | -0.4467 to -0.1533 | Yes | **** | <0.0001 |
| Sav-H vs. Sav-UF3P | -0,52 | -0.6667 to -0.3733 | Yes | **** | <0.0001 |
| Sav-UF10R vs. Sav-UF5R | -0,86 | -1.007 to -0.7133 | Yes | **** | <0.0001 |
| Sav-UF10R vs. Sav-UF3R | -1,57 | -1.717 to -1.423 | Yes | **** | <0.0001 |
| Sav-UF10R vs. Sav-UF3P | -1,79 | -1.937 to -1.643 | Yes | **** | <0.0001 |
| Sav-UF5R vs. Sav-UF3R | -0,71 | -0.8567 to -0.5633 | Yes | **** | <0.0001 |
| Sav-UF5R vs. Sav-UF3P | -0,93 | -1.077 to -0.7833 | Yes | **** | <0.0001 |
| Sav-UF3R vs. Sav-UF3P | -0,22 | -0.3667 to -0.07330 | Yes | *** | 0.0006 |
|  |  |  |  |  |  |
| 50 µg/ml |  |  |  |  |  |
| Sav-H vs. Sav-UF10R | 1,36 | 1.213 to 1.507 | Yes | **** | <0.0001 |
| Sav-H vs. Sav-UF5R | 0,35 | 0.2033 to 0.4967 | Yes | **** | <0.0001 |
| Sav-H vs. Sav-UF3R | -0,33 | -0.4767 to -0.1833 | Yes | **** | <0.0001 |
| Sav-H vs. Sav-UF3P | -0,59 | -0.7367 to -0.4433 | Yes | **** | <0.0001 |
| Sav-UF10R vs. Sav-UF5R | -1,01 | -1.157 to -0.8633 | Yes | **** | <0.0001 |
| Sav-UF10R vs. Sav-UF3R | -1,69 | -1.837 to -1.543 | Yes | **** | <0.0001 |
| Sav-UF10R vs. Sav-UF3P | -1,95 | -2.097 to -1.803 | Yes | **** | <0.0001 |
| Sav-UF5R vs. Sav-UF3R | -0,68 | -0.8267 to -0.5333 | Yes | **** | <0.0001 |
| Sav-UF5R vs. Sav-UF3P | -0,94 | -1.087 to -0.7933 | Yes | **** | <0.0001 |
| Sav-UF3R vs. Sav-UF3P | -0,26 | -0.4067 to -0.1133 | Yes | **** | <0.0001 |
|  |  |  |  |  |  |
| 25 µg/ml |  |  |  |  |  |
| Sav-H vs. Sav-UF10R | 1,27 | 1.123 to 1.417 | Yes | **** | <0.0001 |
| Sav-H vs. Sav-UF5R | 0,56 | 0.4133 to 0.7067 | Yes | **** | <0.0001 |
| Sav-H vs. Sav-UF3R | 0 | -0.1467 to 0.1467 | No | ns | >0.9999 |
| Sav-H vs. Sav-UF3P | -0,1 | -0.2467 to 0.04670 | No | ns | 0.3274 |
| Sav-UF10R vs. Sav-UF5R | -0,71 | -0.8567 to -0.5633 | Yes | **** | <0.0001 |
| Sav-UF10R vs. Sav-UF3R | -1,27 | -1.417 to -1.123 | Yes | **** | <0.0001 |
| Sav-UF10R vs. Sav-UF3P | -1,37 | -1.517 to -1.223 | Yes | **** | <0.0001 |
| Sav-UF5R vs. Sav-UF3R | -0,56 | -0.7067 to -0.4133 | Yes | **** | <0.0001 |
| Sav-UF5R vs. Sav-UF3P | -0,66 | -0.8067 to -0.5133 | Yes | **** | <0.0001 |
| Sav-UF3R vs. Sav-UF3P | -0,1 | -0.2467 to 0.04670 | No | ns | 0.3274 |
|  |  |  |  |  |  |
| 12.5 µg/ml |  |  |  |  |  |
| Sav-H vs. Sav-UF10R | 0,85 | 0.7033 to 0.9967 | Yes | **** | <0.0001 |
| Sav-H vs. Sav-UF5R | 0,51 | 0.3633 to 0.6567 | Yes | **** | <0.0001 |
| Sav-H vs. Sav-UF3R | 0,06 | -0.08670 to 0.2067 | No | ns | 0.7869 |
| Sav-H vs. Sav-UF3P | -0,41 | -0.5567 to -0.2633 | Yes | **** | <0.0001 |
| Sav-UF10R vs. Sav-UF5R | -0,34 | -0.4867 to -0.1933 | Yes | **** | <0.0001 |
| Sav-UF10R vs. Sav-UF3R | -0,79 | -0.9367 to -0.6433 | Yes | **** | <0.0001 |
| Sav-UF10R vs. Sav-UF3P | -1,26 | -1.407 to -1.113 | Yes | **** | <0.0001 |
| Sav-UF5R vs. Sav-UF3R | -0,45 | -0.5967 to -0.3033 | Yes | **** | <0.0001 |
| Sav-UF5R vs. Sav-UF3P | -0,92 | -1.067 to -0.7733 | Yes | **** | <0.0001 |
| Sav-UF3R vs. Sav-UF3P | -0,47 | -0.6167 to -0.3233 | Yes | **** | <0.0001 |

**Table S8** – Statistical analysis of ACEi activity assay results for Alcalase total hydrolysate (Alc-H) and its peptide fractions of varying molecular weights (Alc-UF10R, Alc-UF5R, Alc-UF3R, Alc-UF3P), performed using two-way ANOVA followed by Tukey’s multiple comparison test

| Table Analyzed | Alcalase ACEi activity of hydrolysate and peptide fractions |
| --- | --- |

| Two-way ANOVA | Ordinary |
| --- | --- |
| Alpha | 0,05 |

| Source of Variation | % of total variation | P value | P value summary | Significant? |  |
| --- | --- | --- | --- | --- | --- |
| Interaction | 0,9532 | 0,5523 | ns | No |  |
| Row Factor | 58,59 | <0.0001 | **** | Yes |  |
| Column Factor | 31,61 | <0.0001 | **** | Yes |  |
|  |  |  |  |  |  |
| ANOVA table | SS | DF | MS | F (DFn, DFd) | P value |
| Interaction | 198,8 | 12 | 16,57 | F (12, 100) = 0.8974 | P=0.5523 |
| Row Factor | 12221 | 4 | 3055 | F (4, 100) = 165.5 | P<0.0001 |
| Column Factor | 6593 | 3 | 2198 | F (3, 100) = 119.0 | P<0.0001 |
| Residual | 1846 | 100 | 18,46 |  |  |
|  |  |  |  |  |  |
| Data summary |  |  |  |  |  |
| Number of columns (Column Factor) | 4 |  |  |  |  |
| Number of rows (Row Factor) | 5 |  |  |  |  |
| Number of values | 120 |  |  |  |  |

Multiple comparison

Within each column, compare rows (simple effects within columns)

| Number of families | 4 |
| --- | --- |
| Number of comparisons per family | 10 |
| Alpha | 0,05 |

| Tukey's multiple comparisons test | Mean Diff. | 95.00% CI of diff. | Below  threshold? | Summary | Adjusted P  Value |
| --- | --- | --- | --- | --- | --- |
|  |  |  |  |  |  |
| 100 µg/ml |  |  |  |  |  |
| Alc-H vs. Alc-UF10R | 24,02 | 17.13 to 30.91 | Yes | **** | <0.0001 |
| Alc-H vs. Alc-UF5R | 15,91 | 9.018 to 22.80 | Yes | **** | <0.0001 |
| Alc-H vs. Alc-UF3R | 7,35 | 0.4578 to 14.24 | Yes | * | 0,0305 |
| Alc-H vs. Alc-UF3P | -8,56 | -15.45 to -1.668 | Yes | ** | 0,0072 |
| Alc-UF10R vs. Alc-UF5R | -8,11 | -15.00 to -1.218 | Yes | * | 0,0126 |
| Alc-UF10R vs. Alc-UF3R | -16,67 | -23.56 to -9.778 | Yes | **** | <0.0001 |
| Alc-UF10R vs. Alc-UF3P | -32,58 | -39.47 to -25.69 | Yes | **** | <0.0001 |
| Alc-UF5R vs. Alc-UF3R | -8,56 | -15.45 to -1.668 | Yes | ** | 0,0072 |
| Alc-UF5R vs. Alc-UF3P | -24,47 | -31.36 to -17.58 | Yes | **** | <0.0001 |
| Alc-UF3R vs. Alc-UF3P | -15,91 | -22.80 to -9.018 | Yes | **** | <0.0001 |
|  |  |  |  |  |  |
| 50 µg/ml |  |  |  |  |  |
| Alc-H vs. Alc-UF10R | 22,58 | 15.69 to 29.47 | Yes | **** | <0.0001 |
| Alc-H vs. Alc-UF5R | 14,96 | 8.063 to 21.85 | Yes | **** | <0.0001 |
| Alc-H vs. Alc-UF3R | 6,909 | 0.01681 to 13.80 | Yes | * | 0,0491 |
| Alc-H vs. Alc-UF3P | -8,046 | -14.94 to -1.154 | Yes | * | 0,0136 |
| Alc-UF10R vs. Alc-UF5R | -7,623 | -14.52 to -0.7312 | Yes | * | 0,0224 |
| Alc-UF10R vs. Alc-UF3R | -15,67 | -22.56 to -8.778 | Yes | **** | <0.0001 |
| Alc-UF10R vs. Alc-UF3P | -30,63 | -37.52 to -23.73 | Yes | **** | <0.0001 |
| Alc-UF5R vs. Alc-UF3R | -8,046 | -14.94 to -1.154 | Yes | * | 0,0136 |
| Alc-UF5R vs. Alc-UF3P | -23 | -29.89 to -16.11 | Yes | **** | <0.0001 |
| Alc-UF3R vs. Alc-UF3P | -14,96 | -21.85 to -8.063 | Yes | **** | <0.0001 |
|  |  |  |  |  |  |
| 25 µg/ml |  |  |  |  |  |
| Alc-H vs. Alc-UF10R | 21,14 | 14.25 to 28.03 | Yes | **** | <0.0001 |
| Alc-H vs. Alc-UF5R | 14 | 7.109 to 20.89 | Yes | **** | <0.0001 |
| Alc-H vs. Alc-UF3R | 6,468 | -0.4242 to 13.36 | No | ns | 0,0767 |
| Alc-H vs. Alc-UF3P | -7,533 | -14.42 to -0.6406 | Yes | * | 0,0249 |
| Alc-UF10R vs. Alc-UF5R | -7,137 | -14.03 to -0.2446 | Yes | * | 0,0386 |
| Alc-UF10R vs. Alc-UF3R | -14,67 | -21.56 to -7.777 | Yes | **** | <0.0001 |
| Alc-UF10R vs. Alc-UF3P | -28,67 | -35.56 to -21.78 | Yes | **** | <0.0001 |
| Alc-UF5R vs. Alc-UF3R | -7,533 | -14.42 to -0.6406 | Yes | * | 0,0249 |
| Alc-UF5R vs. Alc-UF3P | -21,53 | -28.43 to -14.64 | Yes | **** | <0.0001 |
| Alc-UF3R vs. Alc-UF3P | -14 | -20.89 to -7.109 | Yes | **** | <0.0001 |
|  |  |  |  |  |  |
| 12.5 µg/ml |  |  |  |  |  |
| Alc-H vs. Alc-UF10R | 16,81 | 9.922 to 23.71 | Yes | **** | <0.0001 |
| Alc-H vs. Alc-UF5R | 11,14 | 4.245 to 18.03 | Yes | *** | 0,0002 |
| Alc-H vs. Alc-UF3R | 5,145 | -1.747 to 12.04 | No | ns | 0,2396 |
| Alc-H vs. Alc-UF3P | -5,992 | -12.88 to 0.9002 | No | ns | 0,1197 |
| Alc-UF10R vs. Alc-UF5R | -5,677 | -12.57 to 1.215 | No | ns | 0,1573 |
| Alc-UF10R vs. Alc-UF3R | -11,67 | -18.56 to -4.777 | Yes | **** | <0.0001 |
| Alc-UF10R vs. Alc-UF3P | -22,81 | -29.70 to -15.91 | Yes | **** | <0.0001 |
| Alc-UF5R vs. Alc-UF3R | -5,992 | -12.88 to 0.9002 | No | ns | 0,1197 |
| Alc-UF5R vs. Alc-UF3P | -17,13 | -24.02 to -10.24 | Yes | **** | <0.0001 |
| Alc-UF3R vs. Alc-UF3P | -11,14 | -18.03 to -4.245 | Yes | *** | 0,0002 |

**Table S9** – Statistical analysis of ACEi activity assay results for Savinase total hydrolysate (Sav-H) and its peptide fractions of varying molecular weights (Sav-UF10R, Sav-UF5R, Sav-UF3R, Sav-UF3P), performed using two-way ANOVA followed by Tukey’s multiple comparison test

| Table Analyzed | Savinase ACEi activity of hydrolysate and peptide fractions |
| --- | --- |

| Two-way ANOVA | Ordinary |
| --- | --- |
| Alpha | 0,05 |

| Source of Variation | % of total variation | P value | P value summary | Significant? |  |
| --- | --- | --- | --- | --- | --- |
| Interaction | 1,137 | 0,3109 | ns | No |  |
| Row Factor | 53,09 | <0.0001 | **** | Yes |  |
| Column Factor | 37,71 | <0.0001 | **** | Yes |  |
|  |  |  |  |  |  |
| ANOVA table | SS | DF | MS | F (DFn, DFd) | P value |
| Interaction | 169,3 | 12 | 14,1 | F (12, 100) = 1.176 | P=0.3109 |
| Row Factor | 7901 | 4 | 1975 | F (4, 100) = 164.6 | P<0.0001 |
| Column Factor | 5612 | 3 | 1871 | F (3, 100) = 155.9 | P<0.0001 |
| Residual | 1200 | 100 | 12 |  |  |
|  |  |  |  |  |  |
| Data summary |  |  |  |  |  |
| Number of columns (Column Factor) | 4 |  |  |  |  |
| Number of rows (Row Factor) | 5 |  |  |  |  |
| Number of values | 120 |  |  |  |  |

Multiple Comparison

Within each column, compare rows (simple effects within columns)

| Number of families | 4 |
| --- | --- |
| Number of comparisons per family | 10 |
| Alpha | 0,05 |

| Tukey's multiple comparisons test | Mean Diff. | 95.00% CI of diff. | Below  threshold? | Summary | Adjusted P  Value |
| --- | --- | --- | --- | --- | --- |
|  |  |  |  |  |  |
| 100 µg/ml |  |  |  |  |  |
| Sav-H vs. Sav-UF10R | 19,94 | 14.38 to 25.49 | Yes | **** | <0.0001 |
| Sav-H vs. Sav-UF5R | 13,21 | 7.650 to 18.76 | Yes | **** | <0.0001 |
| Sav-H vs. Sav-UF3R | 6,101 | 0.5447 to 11.66 | Yes | * | 0,0239 |
| Sav-H vs. Sav-UF3P | -7,105 | -12.66 to -1.549 | Yes | ** | 0,0052 |
| Sav-UF10R vs. Sav-UF5R | -6,731 | -12.29 to -1.176 | Yes | ** | 0,0094 |
| Sav-UF10R vs. Sav-UF3R | -13,84 | -19.39 to -8.280 | Yes | **** | <0.0001 |
| Sav-UF10R vs. Sav-UF3P | -27,04 | -32.60 to -21.49 | Yes | **** | <0.0001 |
| Sav-UF5R vs. Sav-UF3R | -7,105 | -12.66 to -1.549 | Yes | ** | 0,0052 |
| Sav-UF5R vs. Sav-UF3P | -20,31 | -25.87 to -14.75 | Yes | **** | <0.0001 |
| Sav-UF3R vs. Sav-UF3P | -13,21 | -18.76 to -7.650 | Yes | **** | <0.0001 |
|  |  |  |  |  |  |
| 50 µg/ml |  |  |  |  |  |
| Sav-H vs. Sav-UF10R | 18,14 | 12.59 to 23.70 | Yes | **** | <0.0001 |
| Sav-H vs. Sav-UF5R | 12,02 | 6.461 to 17.57 | Yes | **** | <0.0001 |
| Sav-H vs. Sav-UF3R | 5,551 | -0.004325 to 11.11 | No | ns | 0,0503 |
| Sav-H vs. Sav-UF3P | -6,465 | -12.02 to -0.9096 | Yes | * | 0,014 |
| Sav-UF10R vs. Sav-UF5R | -6,125 | -11.68 to -0.5697 | Yes | * | 0,0231 |
| Sav-UF10R vs. Sav-UF3R | -12,59 | -18.15 to -7.035 | Yes | **** | <0.0001 |
| Sav-UF10R vs. Sav-UF3P | -24,61 | -30.16 to -19.05 | Yes | **** | <0.0001 |
| Sav-UF5R vs. Sav-UF3R | -6,465 | -12.02 to -0.9096 | Yes | * | 0,014 |
| Sav-UF5R vs. Sav-UF3P | -18,48 | -24.04 to -12.93 | Yes | **** | <0.0001 |
| Sav-UF3R vs. Sav-UF3P | -12,02 | -17.57 to -6.461 | Yes | **** | <0.0001 |
|  |  |  |  |  |  |
| 25 µg/ml |  |  |  |  |  |
| Sav-H vs. Sav-UF10R | 16,75 | 11.19 to 22.30 | Yes | **** | <0.0001 |
| Sav-H vs. Sav-UF5R | 11,09 | 5.537 to 16.65 | Yes | **** | <0.0001 |
| Sav-H vs. Sav-UF3R | 5,124 | -0.4314 to 10.68 | No | ns | 0,0854 |
| Sav-H vs. Sav-UF3P | -5,968 | -11.52 to -0.4123 | Yes | * | 0,0288 |
| Sav-UF10R vs. Sav-UF5R | -5,654 | -11.21 to -0.09851 | Yes | * | 0,044 |
| Sav-UF10R vs. Sav-UF3R | -11,62 | -17.18 to -6.067 | Yes | **** | <0.0001 |
| Sav-UF10R vs. Sav-UF3P | -22,71 | -28.27 to -17.16 | Yes | **** | <0.0001 |
| Sav-UF5R vs. Sav-UF3R | -5,968 | -11.52 to -0.4123 | Yes | * | 0,0288 |
| Sav-UF5R vs. Sav-UF3P | -17,06 | -22.62 to -11.50 | Yes | **** | <0.0001 |
| Sav-UF3R vs. Sav-UF3P | -11,09 | -16.65 to -5.537 | Yes | **** | <0.0001 |
|  |  |  |  |  |  |
| 12.5 µg/ml |  |  |  |  |  |
| Sav-H vs. Sav-UF10R | 13,16 | 7.602 to 18.71 | Yes | **** | <0.0001 |
| Sav-H vs. Sav-UF5R | 8,715 | 3.160 to 14.27 | Yes | *** | 0,0003 |
| Sav-H vs. Sav-UF3R | 4,026 | -1.529 to 9.582 | No | ns | 0,2672 |
| Sav-H vs. Sav-UF3P | -4,689 | -10.24 to 0.8666 | No | ns | 0,1396 |
| Sav-UF10R vs. Sav-UF5R | -4,443 | -9.998 to 1.113 | No | ns | 0,1803 |
| Sav-UF10R vs. Sav-UF3R | -9,132 | -14.69 to -3.576 | Yes | *** | 0,0001 |
| Sav-UF10R vs. Sav-UF3P | -17,85 | -23.40 to -12.29 | Yes | **** | <0.0001 |
| Sav-UF5R vs. Sav-UF3R | -4,689 | -10.24 to 0.8666 | No | ns | 0,1396 |
| Sav-UF5R vs. Sav-UF3P | -13,4 | -18.96 to -7.849 | Yes | **** | <0.0001 |
| Sav-UF3R vs. Sav-UF3P | -8,715 | -14.27 to -3.160 | Yes | *** | 0,0003 |
